# Treating ICB-resistant cancer by inhibiting PD-L1 via DHHC3 degradation induced by cell penetrating peptide-induced chimera conjugates

**DOI:** 10.1038/s41419-024-07073-y

**Published:** 2024-09-30

**Authors:** Yu-Ying Shi, Gang Fan, Ruirong Tan, Shan Li, Hua-Bing Sun, Rui Li, Mengni Yang, Shanshan Gao, Miao Liu, Meng-Yuan Dai

**Affiliations:** 1 Department of Gynecological Oncology, Zhongnan Hospital of Wuhan University, Wuhan, China; 2 Department of Urology, Huazhong University of Science and Technology Union Shenzhen Hospital, Shenzhen, China; 3 ChinaTranslational Chinese Medicine Key Laboratory of Sichuan Province, State Key Laboratory of Quality Evaluation of Traditional Chinese Medicine, Sichuan Institute for Translational Chinese Medicine, Sichuan Academy of Chinese Medicine Sciences, Chengdu, China; 4 Tianjin Key Laboratory on Technologies Enabling Development of Clinical Therapeutics and Diagnostics, School of Pharmacy, Department of Nuclear Medicine, Tianjin Medical University General Hospital, Tianjin Medical University, Tianjin, China; 5 Department of Radiation Oncology, Radiation Oncology Key Laboratory of Sichuan Province, Sichuan Clinical Research Center for Cancer, Sichuan Cancer Hospital and Institute, Sichuan Cancer Center, Chengdu, China; 6 Department of Biomedical Sciences, Faculty of Health Sciences, University of Macau, Taipa, Macau, China; 7 Department of Pathology, Brigham and Women’s Hospital, Harvard Medical School, Boston, MA USA

**Keywords:** Immune cell death, Tumour biomarkers

## Abstract

The current selection of ligands for both proteins of interest (POI) and E3 ubiquitin ligase significantly restricts the scope of targeted protein degradation (TPD) technologies. This study introduces cell-penetrating peptide-induced chimera conjugates (cp-PCCs) targeting the DHHC3 enzyme involved in PD-L1 palmitoylation. This approach disrupts PD-L1’s immunosuppressive function, enhancing anti-tumor immunity. We developed cp-PCCs to degrade DHHC3, directly linking DHHC3-mediated PD-L1 palmitoylation to PD-L1 stability on tumor cells. Our research utilized both in vitro assays and in vivo experiments in immune checkpoint blockade-resistant mouse models. We focused on a CRBN-based cp-PCC named PCC16, which demonstrated a DC50 of 102 nmol for DHHC3 degradation and significantly reduced PD-L1 levels. In resistant models, PCC16 not only robustly downregulated PD-L1 but also exhibited substantial anti-tumor activity in vivo without significant toxicity. This outperformed traditional inhibitors, showcasing the potential of cp-PCC technology to bypass current PROTAC limitations. Our findings suggest that cp-PCCs offer a promising method for targeting PD-L1 through DHHC3 inhibition and support their continued exploration as a versatile tool in cancer immunotherapy, especially for tumors resistant to standard treatments.

## Introduction

Recent advances in oncology highlight the crucial role of immune checkpoint blockers (ICBs) in targeting programmed death-1 (PD-1) receptors and their ligand PD-L1 [1–3]. They counteract tumor-mediated immunosuppression by activating T cells against tumor cells [4–6]. Despite clinical success, variability in patient response and development of resistance pose significant challenges that are potentially linked to the tumor microenvironment, mutation burden, PD-L1 expression, and individual immune status [7–10]. Resistance varies across cancer types and patient populations. In melanoma [11] and head and neck squamous cell carcinoma (HNSC) [12], high initial responses to PD-1/PD-L1 inhibitors are observed, but resistance develops in several patients with different types of cancer [8, 13]. In cervical cancer, approximately one-third of squamous cell carcinoma cases and one-sixth of adenocarcinoma cases exhibit PD-L1 expression, suggesting potential treatment resistance or nonresponsiveness in some patients [14]. Elevated PD-L1 expression does not mitigate the risk of resistance development. In triple-negative breast cancer, which is known for its aggressiveness and nonresponsiveness to standard treatments, the efficacy of PD-1/PD-L1 inhibitors is limited [9, 15]. To address resistance, the strategy combining PD-1/PD-L1 inhibitors with cytotoxic agents, radiation treatment, or various additional immunotherapeutic approaches is being investigated to improve therapeutic outcomes [16]. Deeper exploration of the PD-1/PD-L1 pathway will aid in the development of advanced immunotherapies.

Targeted protein degradation (TPD) is a pioneering approach for developing specific degraders that target the proteins of interest (POIs) [17]. Within this realm, proteolysis-targeting chimeras (PROTACs) stand out, with several candidates advancing through clinical trials [18–21]. They are dual-functional molecules composed of two moieties: a POI binder and an E3 ligase binder connected via a chemical linker. This method, which is distinct from conventional inhibitors, facilitates POI degradation via the ubiquitin-proteasome pathway [22]. Such innovative strategies offer significant advantages in tumor treatment, particularly in countering drug resistance and enhancing the scope of targetable proteins in cancer therapies. Currently, most PROTACs are small molecule-based structures, and their development encounters challenges, particularly in identifying small molecule ligands that can target POIs. This is attributed to the lack of suitable binding sites in most protein structures [23, 24]. In contrast, peptides are effective in specifically targeting protein-protein interactions, frequently exhibiting a remarkable degree of specificity and affinity [25]. The first PROTAC molecule, termed Protac-1, comprises two parts: ovalicin, targeting METAP2, and a peptide derived from nuclear factor-κB inhibitor-α for binding to β-transducin repeat-containing E3 ubiquitin–protein ligase [26]. However, limitations arise owing to difficulties in cell membrane penetration and the absence of efficient small-molecule E3 ligase ligands. Although more than 600 E3 ubiquitin ligases have been identified, only a few corresponding E3 ubiquitin ligase ligands have been developed. Specifically, a few ligands have been used in designing PROTAC molecules, such as Von-Hippel-Lindau (VHL), Cereblon (CRBN), Mouse Double Minute 2 homolog, and Inhibitor of Apoptosis Protein (IAP). Notably, ligases such as VHL, CRBN, and IAP have received high confidence scores due to extensive knowledge regarding them in terms of functionality and substrate [27]. Importantly, PROTACs related to VHL and CRBN have progressed to the clinical trial stages [28]. In our previous research, we employed PROTAC molecules composed entirely of peptides. While this approach allowed for the design of peptide structures with relatively strong binding affinities towards the POIs, the selection for linking to E3 ubiquitin ligases was significantly limited, complicating the linkage to some of the more efficient E3 ubiquitin ligases. Therefore, combining peptide ligands targeting the POIs with high-efficiency small molecule ligands targeting E3 ubiquitin ligases could potentially represent a more favorable modality [29, 30].

Peptide degraders generally face difficulties entering cells, primarily because of their low membrane permeability. Nonetheless, in recent years, various techniques and strategies have been developed to enhance the intracellular uptake efficiency of peptides. cell-penetrating peptides (CPPs) are a class of short peptides capable of entering cells through multiple pathways. They achieve cellular internalization by interacting with the cell surface or several other modes. These peptides facilitate the entry of loaded proteins or other macromolecules into cells through direct interactions with the cell membrane or mechanisms that promote endocytosis [31, 32]. The TAT peptide is a well-known CPP used extensively in biomedical research, particularly for drug delivery and gene therapy [33, 34]. TAT peptides, derived from the transactivating transcriptional activator protein of the Human Immunodeficiency Virus, have garnered significant attention for their efficient cellular delivery capabilities [35, 36]. The typical sequence of TAT peptide, “YGRKKRRQRRR,” contains multiple arginine residues, aiding in its ability to cross cell membranes [31, 37]. The positively charged nature of this peptide is crucial for its efficient membrane permeability.

Although PROTACs are promising in terms of degradation of membrane proteins, the technology is still in its initial stages and can only be considered an active area of research and development [38]. Intracellular factors that play significant roles in the PD-1/PD-L1 pathway are excellent targets for current TPD technologies. Palmitoyltransferases, particularly DHHC3 (ZDHHC3), are crucial for PD-L1 palmitoylation. DHHC3 post-translationally modifies proteins by attaching palmitic acid to specific cysteine residues, thereby affecting protein stability, lipophilicity, and localization [39]. Such modifications of PD-L1 by DHHC3 enhance its membrane anchoring and aggregation, potentially increasing T cell PD-1 interaction, and thereby, immunosuppression. PD-L1 expressed on the cell membrane, especially in its unpalmitoylated or unstable form, is subject to internalization and subsequent lysosomal degradation [40]. Palmitoylation by DHHC3 diminishes internalization and lysosomal degradation, thereby prolonging the presence of PD-L1 on the cell surface and its associated immunosuppressive effects [41]. Consequently, targeting DHHC3 to decrease PD-L1 palmitoylation and membrane residence is posited as a potential antitumor strategy aimed at enhancing the immune response against tumors.

In recent years, non-small-molecule degraders have received considerable attention [42]. Here, we investigated the role of the palmitoyltransferase DHHC3 in inhibiting PD-L1 expression using the TPD method. We developed an innovative non-small-molecule modality that intricately combines membrane-permeable peptide structures with peptides that specifically target DHHC3. The combined peptides were further conjugated with small molecule E3 ubiquitin ligase ligands to form cell-penetrating peptide-induced chimera conjugates (cp-PCCs). The principal objective of our study was to evaluate the potential effectiveness of the PCC degrader modality in addressing ICB resistance, which is a significant hurdle in tumor therapy.

## Results

### Design and preliminary efficacy of cp-PCC targeting palmitoylation and expression of PD-L1

**Fig. 1:**
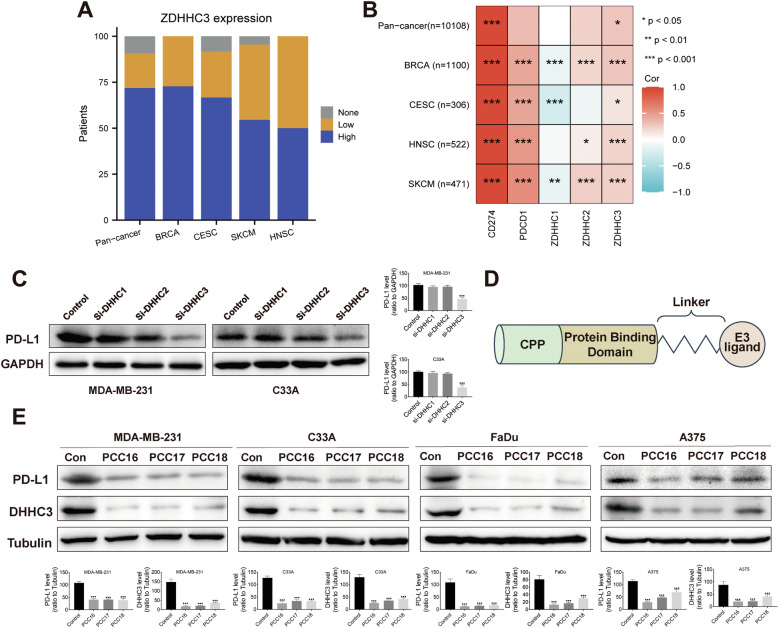
Preliminary effectiveness of cp-PCC compounds aimed at the palmitoylation and production of PD-L1. **A** The expression of ZDHHC3 protein in cancers based on the Human Protein Atlas: ZDHHC3 expression levels are evaluated according to the immunohistochemistry staining on cancer tissue samples of the Human Protein Atlas database. The X axis represents the cancer type. Pan-cancer includes 20 different cancer types. BRCA, breast cancer. CESC, cervical cancer. SKCM, skin cancer and melanoma. HNSC, head and neck cancer. The Y axis represents the percentage of patients with specific expression pattern of ZDHHC3. High, high expression. Low, low expression. None, no expression. **B** The correlation between PDL1 (CD274) and PD1(PDCD1), ZDHHC1, ZDHHC2, or ZDHHC3 expression in TCGA cancer types. TIMER2.0 Gene_Corr Module is used to evaluate the expression between two genes in different cancer types of TCGA database. The heatmap lists the purity-adjusted partial spearman’s rho (Cor) value as the degree of the correlation between two genes and the number (n) of patient cases in each cancer typer. **C** Western blot analysis was utilized to examine the expression of PD-L1 protein in MDA-MB-231 and C33A cells following the knockdown of DHHC1, DHHC2, and DHHC3. **D** A schematic representation of the structural design of cp-PCC. **E** Western blot analysis was conducted to assess the expression of PD-L1 protein in four cell lines (MDA-MB-231, C33A, FaDu, A375) following a 6-hour incubation with 10 µM PCC16/17/18, each corresponding to CRBN/VHL/IAP-based cp-PCC, respectively. Quantitative data are presented as mean ± standard error. Statistical analysis was performed using one-way ANOVA with Tukey’s post hoc test (*n* = 3): *P < 0.05, **P < 0.01, ***P < 0.001.

The ZDHHC3 protein is expressed in all breast cancer (BRCA) and HNSCpatients, and in almost all pan-cancer, cervical cancer (CES), and skin cancer and melanoma (SKCM) patients based on immunohistochemistry staining examination results in the Human Protein Atlas database (Fig. 1A). Regardless of the type of cancer, more than half of the patients exhibit high expression levels of the ZDHH3 protein (Fig. 1A). Gene correlations between PD-L1 and ZDHHC3, PD-1, ZDHHC1, and ZDHHC2 in different cancer samples from the TCGA database are shown in Fig. 1B. PD-L1 and ZDHHC3 were significantly and positively correlated in pan-cancer, BRCA, CES, HNSC, and SKCM patients (Fig. 1B).

Western Blot results indicated that downregulation of DHHC1 and DHHC2 did not significantly alter the expression of PD-L1. However, a marked reduction in PD-L1 expression was observed after DHHC3 knockdown (*P* < 0.05) (Fig. 1C). These data reveal a robust global association between the key immune checkpoint ligand PD-L1 and the lipid enzyme DHHC3 across cancer types. Based on the aforementioned theoretical framework, we designed and synthesized cp-PCC. The cp-PCC is composed of CPPs, a protein-binding domain peptide sequence, a small-molecule linker, and a small-molecule E3 ubiquitin ligase ligand (Fig. 1D). We employed the TAT sequence as the CPP, linking it to a protein-binding domain sequence that has been previously validated for the effective targeting of DHHC3 [43]. The assembly was connected via a polyethylene glycol linker. In this study, human breast cancer cells (MDA-MB-231), human cervical cancer cells (C33A), human head and neck squamous carcinoma cells (FaDu), and human malignant melanoma cells (A375) were treated with 10 µM of PCC series drugs. Following a 6-h incubation period, Western Blot analysis was conducted to assess the levels of PD-L1 protein after treatment. The results indicated that, compared to the control group, the degradation of the PD-L1 protein was observed in all four cell lines treated with PCC16/17/18, with substantial statistical significance compared to the control (*P* < 0.05) (Fig. 1E).

### cp-PCCs significantly decrease PD-L1 protein in cell lines

**Fig. 2:**
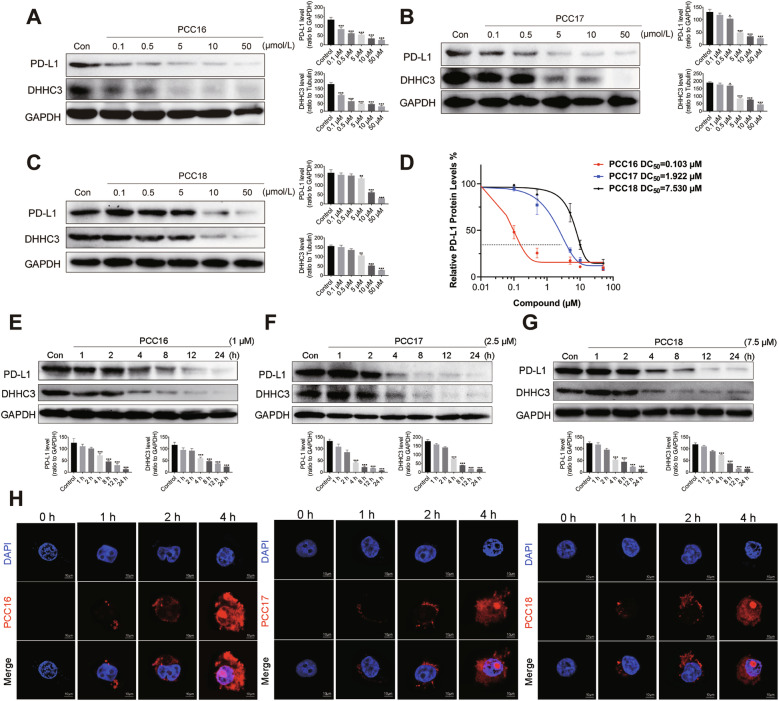
The effect of cp-PCCs in reducing PD-L1 protein in vitro. **A**–**C** Western blot analysis was utilized to examine the expression of DHHC3/PD-L1 proteins in C33A cells following 6 h of treatment with varying concentrations of PCC16/17/18. **D** Comparative analysis of the DC50 values for PCC16/17/18. **E**–**G** Western blot analysis to assess the expression of DHHC3/PD-L1 proteins in C33A cells post-incubation with specific concentrations of PCC16/17/18 for different time periods. **H** Confocal microscopy was employed to observe the fluorescence intensity of PCC16/17/18 entering the cells at various time points. Quantitative data are presented as mean ± standard error. Statistical analysis was performed using one-way ANOVA with Tukey’s post hoc test (*n* = 3): *P < 0.05, **P < 0.01, ***P < 0.001.

We tested three small-molecule E3 ubiquitin ligase ligands: CRBN, VHL, and IAP. The cp-PCCs linked to these three ligands were designated as PCC16, PCC17, and PCC18, respectively. To investigate the degradation levels of DHHC3 and PD-L1 proteins by the PCC16/17/18 series of drugs, we initially treated C33A cells with the drugs at concentrations of 0.1/0.5/5/10/50 µM. Subsequently, we used Western Blot analysis to detect the levels of DHHC3 and PD-L1 proteins following treatment with the cp-PCC series of drugs. The Western Blot results indicated significant degradation of DHHC3 and PD-L1 proteins at 0.1 µM by PCC16 (Fig. 2A), at 5 µM by PCC17 (Fig. 2B), and at 10 µM by PCC18 (Fig. 2C). Additionally, we calculated the DC50 values for the three drugs, with CRBN-based PCC16 exhibiting the best degradation activity among the three (DC50 = 0.103 µM), VHL-based PCC17 with a DC50 of 1.92 2 µM, and IAP-based PCC18 with a DC50 of 7.530 µM (Fig. 2D). Additionally, we incubated the cp-PCC series of drugs with C33A cells at various time points (0, 1, 2, 4, 8, 12, and 24 h). The analysis indicated that a minimum incubation period of 4 h was necessary to achieve significant degradation of the target proteins DHHC3 and PD-L1 (*P* < 0.05) (Fig. 2E–G). Using confocal microscopy, we dynamically monitored drug penetration into the cell membrane at different time intervals. The results revealed a gradual increase in the intracellular accumulation of PCC16, PCC17, and PCC18 with extended incubation time (Fig. 2H). Additionally, in murine cervical cancer cells (U14), colon cancer cells (CT26), and melanoma cells (B16F10), Western Blot analyses were conducted to evaluate the levels of PD-L1 protein after treatment with PCC16 at varying concentrations (0.1, 0.5, 5, 10, and 50 µM). The results indicated a concentration-dependent decrease in PD-L1 protein levels across all cell types (Supplementary Fig. S1). Collectively, these findings demonstrate that the degradation of the target protein DHHC3 and the decrease in PD-L1 induced by the PCC16/17/18 drugs were both time- and concentration-dependent.

### Synthesis and validation of the cp-PCC pharmaceutical compound

**Fig. 3:**
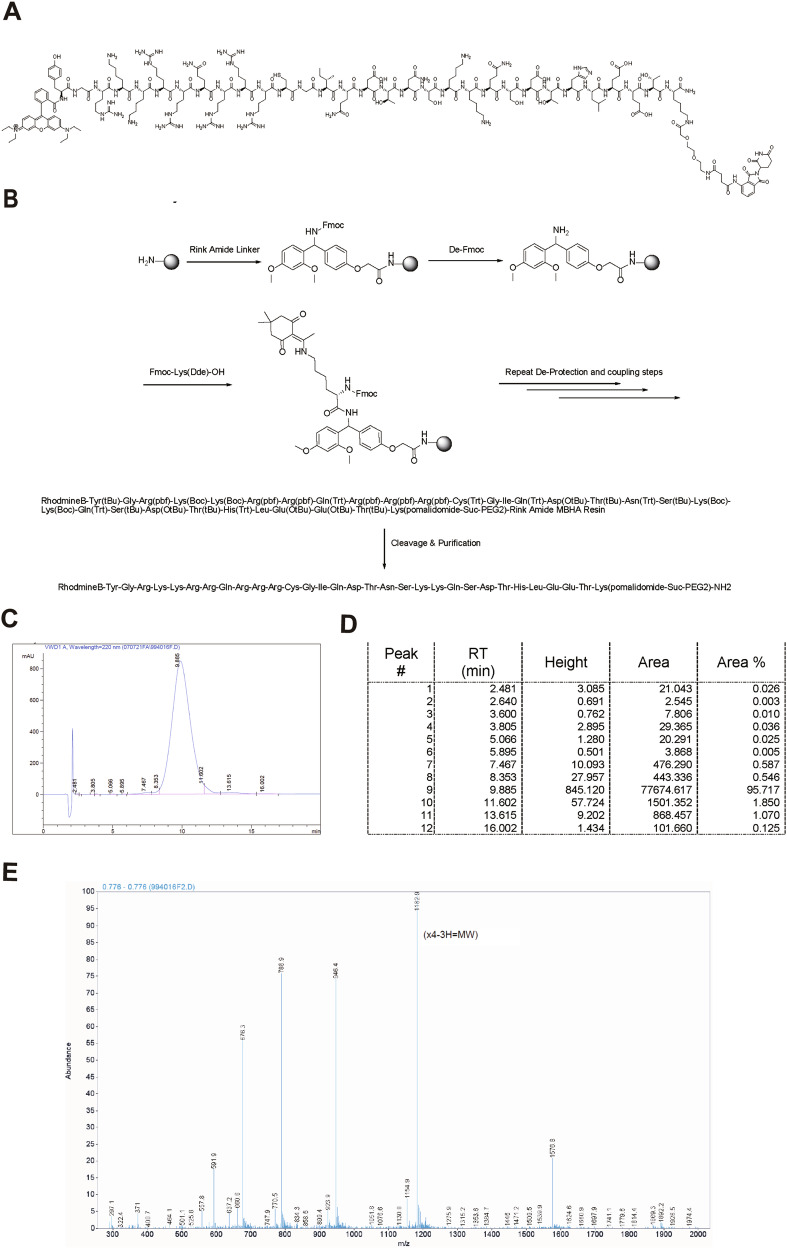
The synthesis and confirmation of the PCC-16. **A** Structure diagram of PCC16 (CRBN-based cp-PCC). **B** Synthetic Route of PCC16. **C** the area under the curve represents the sample’s absorbance at 220 nm during the HPLC procedure. **D** the parameters of each peak include RT: Retention Time. Height: The vertical measurement from the baseline to the peak. Area: The integrated area under each distinct peak. % Area: The proportion of the peak’s area relative to the total peak area. **E** Determination of Molecular Weight (MW): The MW calculation is based on the following equation: Actual MW = (Peak Value × Number of Charges) − Number of Positive Charges + Charge of the positive ion (Rhodamine B as the carrier). The calculated theoretical MW is 4729.3, in contrast to the experimentally observed MW of 4728.6.

The structural configuration of the cp-PCC compound is illustrated in Fig. 3A and Supplementary Fig. S2), which is consistent with the rhodamine and TAT groups. The synthesized peptides were purified by HPLC to achieve a purity level exceeding 95%. The synthesis and verification of the compound were performed by the Chinese Peptide Company (Hangzhou, China), and the synthetic route was shown in Fig. 3B. Throughout the purification phase, HPLC was performed for Quality Control (QC). Subsequent to the attainment of QC clearance, the sample underwent lyophilization to yield a solid powder form (Fig. 3C–E, Supplementary Fig. S3).

### PCC16 exhibited stable binding affinity to the target protein DHHC3

**Fig. 4:**
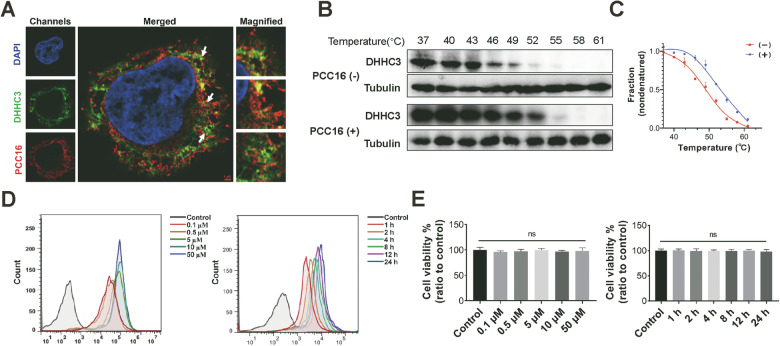
PCC16 demonstrated stable binding affinity to the target protein DHHC3. **A** Confocal observation of DHHC3 binding with drug PCC16. Scale bar, 1 µM. **B** C33A cell lysates treated at different temperatures after incubation with 0.5 µM PCC16, with Western blot analysis of DHHC3 protein expression; **C** CETSA melting curve plotted. **D** Flow cytometry analysis of fluorescent cells after treatment with Rhodamine-labeled PCC16 at different concentration gradients; **E** C33A cells treated with PCC16 over time and concentration gradients, with cell toxicity of PCC16 measured by MTT assay.

In this study, after incubating C33A cells with 2.5 µM of PCC16 for 6 h, high-resolution confocal microscopy revealed the colocalization of the DHHC3 protein and PCC16 (Fig. 4A), suggesting potential effective binding between the drug and the target protein DHHC3. Further analysis of the target specificity of PCC16 in C33A cells was conducted using the cellular thermal shift assay. Cells, with or without PCC16 treatment, were heated at 37–61 °C for a specific duration, followed by Western Blot analysis. The results indicated that, in the untreated group, significant degradation of DHHC3 occurred at 49 °C; however, in the PCC16-treated cells, DHHC3 was largely degraded only at 55 °C (Fig. 4B). The temperature-protein expression curves plotted from these data showed a slower decline in DHHC3 protein levels with increasing temperature in the treated group, with a noticeable right shift in the melting curve (Fig. 4C). These results suggest that PCC16 effectively binds to DHHC3, thereby altering its thermal stability. Additionally, the cellular uptake of the drug by C33A cells was quantified using flow cytometry to measure the fluorescence intensity. As the treatment duration and drug concentration increased, a gradual increase in cells exhibiting red fluorescence was observed, indicating the presence of PCC16 (Fig. 4D). To assess the cytotoxic effects of PCC16 on C33A cells, the MTT assay was conducted to monitor the changes in cell numbers across different times and concentration gradients. Even after incubating the cells for up to 24 h or at concentrations as high as 50 µM, the drugs exhibited relatively low cytotoxicity with no significant differences between groups (Fig. 4E).

### Investigation of the degradation mechanism of cp-PCC compounds

**Fig. 5:**
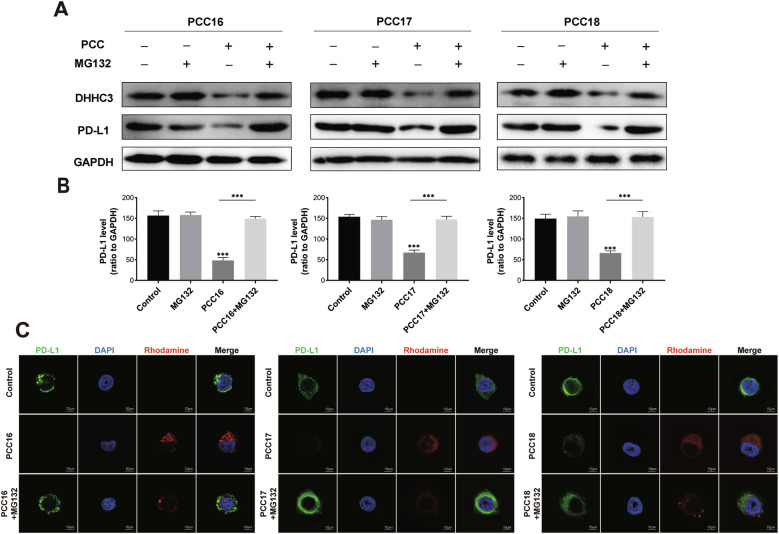
Examination of the degradation mechanism of cp-PCC compounds. **A** C33A cells pre-treated with/without MG132 for 4 h and then treated with PCC16/17/18, with Western blot analysis to detect PD-L1 protein levels; **B** Bar graph representing the quantification of grayscale values; **C** Confocal microscopy observation of the immunofluorescence intensity of PD-L1 protein under PCC16/17/18 ± MG132 conditions. Scale bar, 10 μM. Quantitative data expressed as mean ± standard error; analyzed by one-way ANOVA with Tukey’s post hoc test (*n* = 3): *P < 0.05, **P < 0.01, ***P < 0.001.

We further explored whether the cp-PCC series of drugs enhance the ubiquitination of DHHC3via the proteasome pathway, thereby promoting the degradation of PD-L1. Western Blot results showed that after 6 h of treatment with PCC16/17/18, the protein levels of DHHC3 and PD-L1 decreased. Addition of the proteasome inhibitor MG132 significantly reduced the degradation of DHHC3 and PD-L1 proteins (Fig. 5A, B). Concurrently, immunofluorescence results revealed that the fluorescence intensity of PD-L1 decreased after the addition of PCC16/17/18 and was significantly reversed upon the addition of MG132 (Fig. 5C). These results suggest that PCC16/17/18 can enter cells and inhibit the PD-L1 protein through the proteasome pathway.

### Decrease of PD-L1 via DHHC3 induced by cp-PCCs enhanced chemotherapeutic sensitivity and anti-tumor immunity

**Fig. 6:**
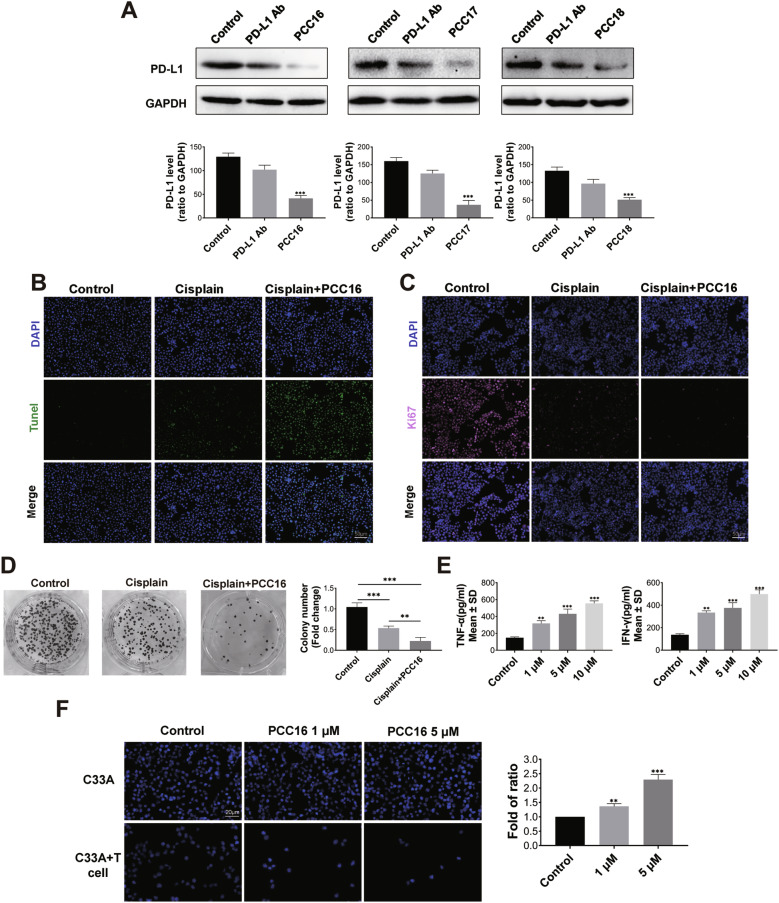
Reduction of PD-L1 through DHHC3 degradation by cp-PCCs increased the sensitivity to chemotherapy and bolstered anti-tumor immunity. **A** Western blot analysis of the effects of PCC series drugs and PD-L1 monoclonal antibody on PD-L1 protein levels in C33A cells; **B** TUNEL assay to assess apoptosis in cervical cancer cells treated with cisplatin and PCC16; **C** Ki67 assay for evaluating the proliferation of cervical cancer cells treated with cisplatin and PCC16; **D** Plate cloning experiment analyzing the proliferation of cervical cancer cells treated with cisplatin and PCC16; **E** In a C33A-T cell co-culture model, ELISA was used to measure IFN-γ and TNF-α expression levels after treatment with different concentrations of PCC16; **F** Hoechest33528 staining in the co-culture system to observe apoptosis of C33A cells after treatment with different concentrations of PCC16. Quantitative data are expressed as mean ± standard error; analyzed by one-way ANOVA with Tukey’s post hoc test (*n* = 3): *P < 0.05, **P < 0.01, ***P < 0.001.

To compare the inhibitory effects of PD-L1 monoclonal antibody and PCC16/17/18 on PD-L1 protein, Western Blot results indicated that at the same concentration, the degradation effect of PCC16/17/18 on C33A cells was significantly superior to that of the PD-L1 monoclonal antibody (Fig. 6A). To investigate whether PCC16 increases the sensitivity of C33A cells to cisplatin, we evaluated the combined effects of cisplatin and PCC16 using TUNEL and Ki67 assays. The results showed that, compared to the control group, cell proliferation decreased and apoptosis increased in the cisplatin group; compared to the cisplatin group alone, the combination of PCC16 and cisplatin significantly reduced proliferation and increased apoptosis, with statistically significant differences (Fig. 6B, C). The colony formation assay, as shown in Fig. 6D, indicated that compared to the control group, the number of C33A colonies decreased in the cisplatin group and significantly decreased in the PCC16 plus cisplatin group, suggesting that PCC16 can enhance the proliferation-inhibitory effects of cisplatin on the C33A cell line. We constructed a T-cell-C33A co-culture system, incubated C33A cells with different concentrations of PCC16 for 6 h, and measured cytokine levels in the supernatant using ELISA. Compared to the control group, PCC16-treated cells significantly increased the secretion of IFN-γ and TNF-α (*P* <0.05) (Fig. 6E). In the co-culture system, after incubation with different concentrations of PCC16 for 6 h, Hoechst 33528 staining was performed to detect the fluorescence intensity of the tumor cells. PCC16 enhanced the T-cell-mediated killing of C33A cells in a concentration-dependent manner (Fig. 6F). These results suggest that PCC16 degrades PD-L1, thereby blocking the interaction between PD-L1 and PD-1, promoting the secretion of IFN-γ and TNF-α into the supernatant of the co-culture system, and ultimately increasing tumor cell apoptosis within the system.

### PCC16 demonstrated efficacious in vitro and in vivo therapeutic effects in combating ICB-resistant tumor

**Fig. 7:**
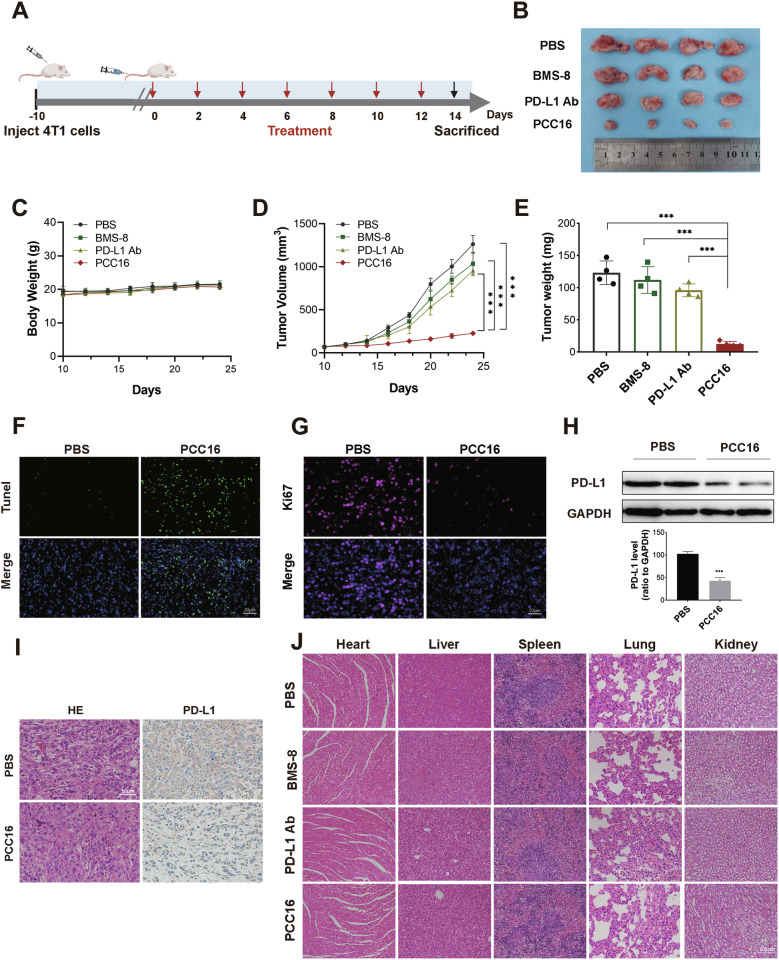
PCC16 showed effective therapeutic results both in vitro and in vivo in treating ICB-resistant tumors. **A** Schematic of treatment regimens in mice with PD-L1 monoclonal antibody, BMS-8, and PCC series drugs; **B** Images of mouse tumors on Day 14 post-treatment; **C** Tumor growth curves for different treatments (*n* = 4); **D** Body weight change curves for mice under different treatments; **E** Comparison of tumor weights in different treatment groups (*n* = 4); **F** Representative tumor sections collected post-treatment (*n* = 4) stained with TUNEL (green) for apoptotic cells, **G** Ki67 (pink) for proliferating cells, and DAPI (blue) for nuclei. **H** Western blot analysis of PD-L1 expression in tumor tissues from each group; **I** H&E and IHC analysis of PD-L1 protein expression post-PCC16 treatment; **J** H&E images of heart, liver, spleen, lung, and kidney tissues from mice in each group; scale bar, 50 μm. Quantitative data expressed as mean ± standard error; analyzed by one-way ANOVA with Tukey’s post hoc test (*n* = 3): *P < 0.05, **P < 0.01, ***P < 0.001.

The 4T1 cell line is a breast cancer cell line derived from mouse mammary tumor tissue and has been demonstrated to be resistant to ICB drugs. To validate the efficacy of cp-PCC drugs in ICB-resistant tumors, we assessed the degradation effect of PCC16 on cellular PD-L1 protein in 4T1 cells using Western Blot analysis after treating the cells with concentration and time gradients. The results demonstrated a significant degradation in PD-L1 protein levels in 4T1 cells under the treatment of PCC16, with substantial statistical significance (*P* < 0.05) (Supplementary Fig. S4). Subsequently, 4T1 cells were injected into the dorsum of BALB/c mice to establish a 4T1 cell xenograft mouse model and study the in vivo therapeutic effects of PCC16. When the tumor volume reached 80–100 mm^3^, the mice were randomly divided into four treatment groups (*n* = 4 each): PBS, PD-L1 inhibitor BMS-8, PD-L1 monoclonal antibody, and PCC16 (Fig. 7A). After 21 days of treatment, tumor growth curves were calculated and plotted based on tumor appearance (Fig. 7B) and growth (Fig. 7C). In the control group, the tumor tissues exhibited the fastest growth and largest volume. Compared to that in the PD-L1 monoclonal antibody and small-molecule PD-L1 inhibitor (BMS-8) groups, PCC16 significantly inhibited tumor growth. Body weight changes in the four groups of mice were not significantly different (Fig. 7D). Tumor weights were measured in each group, and the control group had the largest tumors, whereas the average tumor weights in the BMS-8, PD-L1 monoclonal antibody, and PCC16 groups were 90.8%, 77.9%, and 10.1% of that in the control group, respectively. Compared with the control group, the tumors in the BMS-8 and PD-L1 treatment groups showed a slight decrease in weight and size. However, in the PCC16 treatment group, there was a significant decline in both tumor weight and size compared to the control group, as well as the BMS-8 and PD-L1 monoclonal antibody treatment groups (*P* < 0.05) (Fig. 7C, E). TUNEL staining was used to assess the impact of PCC16 on apoptosis in mouse tumor tissues; compared to the control group, the number of apoptotic tumor cells significantly increased in the PCC16 treatment group (Fig. 7F). Post-PCC16 treatment resulted in notable inhibition of tumor cell proliferation, as demonstrated by anti-ki67 staining (Fig. 7G). Western Blot and immunohistochemistry analyses of randomly collected tumor samples from the PBS and PCC16 groups (Fig. 7H, I) indicated that PCC16 induced PD-L1 protein degradation in vivo. Additionally, histological hematoxylin and eosin staining of the heart, liver, spleen, lungs, and kidneys of mice showed no signs of toxicity (Fig. 7J), indicating good tolerance in all mouse groups throughout the in vivo experiments. These results suggested that PCC16 exhibits strong antitumor activity in vivo.

## Discussion

Resistance to immune checkpoint inhibitors poses a major challenge in both clinical and research settings. Although these drugs offer substantial therapeutic benefits in certain cancer types, the development of drug resistance in many patients significantly reduces their effectiveness [4]. TPD drugs, which function by degrading rather than inhibiting POIs, are a potential approach to overcoming drug resistance [17, 44–46]. However, conclusive studies validating their efficacy in overcoming this challenge are lacking. In our study, the cp-PCC drug targeting DHHC3 degradation aimed at reducing PD-L1 not only efficiently degraded the target protein in high-PD-L1-expressing tumor cell lines but also effectively degraded the target protein in ICB-resistant 4T1 cell lines, causing a significant decrease in PD-L1 expression. More importantly, even when used as a monotherapy in ICB-resistant tumor xenograft experiments, it exerted a substantial therapeutic effect on these resistant tumors. In contrast, PD-L1 monoclonal antibody or small-molecule inhibitor (BMS-8) showed no significant effect on ICB-resistant tumors, either in vitro or in vivo. 4T1 cells expressing PD-L1 demonstrate marked resistance to ICBs. This characteristic renders them an exemplary model for exploring PD-1/PD-L1 targeted therapeutics and elucidating the mechanisms underlying their resistance [47]. In this study, PCC16 exhibited excellent efficacy, providing a promising new direction in the development of immune checkpoint drugs.

Although small-molecule trivalent PROTACs and molecular glues are widely used in TPD, they have significant limitations in terms of the range of target proteins. Typically, the process of identifying small molecule binding sites on target protein structures and developing related small molecule drugs based on these structures is complex. Many target proteins are deemed “undruggable” due to the lack of “deep pocket” structures, which poses a significant challenge in drug discovery [46, 48]. In contrast, peptide PROTACs significantly expand the targeting range by utilizing protein-protein interactions [49]. Current CPP technology has effectively addressed the issue of peptides not easily crossing the cell membrane owing to their large molecular size [31, 32]. However, limited peptide E3 ubiquitin ligase ligands are available. For example, the validated high-affinity ligands of CRL4^CRBN^ E3 ubiquitin ligase are small-molecule structures of immunomodulatory drugs rather than peptide sequences.

Small-molecule drugs targeting specific proteins, such as DHHC3, that can palmitoylate PD-L1 are particularly scarce. Palmitoylation of PD-L1 by DHHC3 leads to the accumulation of PD-L1 on the cell surface, thereby enhancing its ability to inhibit T cells [41]. Thus, palmitoylation of PD-L1 by DHHC3 is crucial for regulating the tumor microenvironment and immune escape mechanisms. In the current study, immunohistochemical staining results from the Human Protein Atlas database revealed that ZDHHC3 is widely expressed in various cancer types. Remarkably, irrespective of the cancer type, more than half of the patients exhibit high ZDHHC3 protein expression levels. In addition, the correlation analysis between PD-L1 and ZDHHC3 across TCGA cancer samples revealed the potential co-expression pattern and positive correlation between PD-L1 and ZDHHC3 in various cancer types. These findings underscore the consistent and widespread association of elevated ZDHHC3 expression across diverse cancer types and suggest a potential crosstalk between ZDHHC3 and PD-L1-dependent immune regulatory pathways, which also highlights the potential therapeutic targeting role of ZDHHC3 in treating different cancers. Theoretically, by inhibiting the activity of DHHC3, palmitoylation and cell surface expression of PD-L1 can be reduced, thereby weakening its inhibitory effect on T cells and enhancing the ability of the immune system to attack tumors. This study provides a potential direction in the development of new cancer immunotherapies. Although some peptide inhibitors exert certain effects, their efficacy is not particularly significant [41]. Our cp-PCC degraders showed considerable potential for the efficient and safe degradation of these targets. In earlier years, peptide-small-molecule E3 ligand chimeric TPD drugs failed to achieve optimal results owing to poor membrane permeability and the constraints of the limited small-molecule E3 ligands availability [26]. Our cp-PCC technology, which targets peptide sequences and guides them into the membrane by CPP, successfully overcomes these limitations. In conjunction with efficient small-molecule E3 ubiquitin ligase ligands, it significantly enhances the degradation efficiency of TPD drugs. In our study, various cp-PCC drugs showed good membrane permeability in multiple cell types and achieved efficient degradation of POIs, thereby significantly reducing PD-L1 expression.

We tested three types of high-efficiency small-molecule E3 ligands linked to peptides. Among them, the CRBN-based cp-PCC, PCC16, demonstrated the most favorable results. It exhibited a DC50 value of approximately 100 nmol for the target protein in C33a cells, which was significantly higher than that of VHL- and IAP-based cp-PCCs. Moreover, PCC16 demonstrated excellent in vivo safety, offering significant potential for clinical translation.

## Conclusions

Our cp-PCC degrader technology is a powerful tool for overcoming ICB resistance. It exhibits tremendous clinical application potential and advantages owing to its highly selective degradation of target proteins, bypassing resistance mechanisms, and excellent in vivo safety.

## Materials and methods

### Pan-cancer analysis of the expression correlation between DHHC3 and PD-L1

To investigate ZDHHC3 protein expression in cancers, we used the pathology section of the Human Protein Atlas (https://www.proteinatlas.org/) database to evaluate the expression of ZDHHC3 protein in 20 different cancer types. The expression levels of ZDHHC3 were summarized based on the results of immunohistochemical staining of cancer tissue sections using an anti-ZDHHC3 antibody. To determine the correlation between gene expression in cancers, we use the Gene_Corr Module of the TIMER2.0 [50] (http://timer.cistrome.org) database to discover the co-expression patterns of PD-L1, PD-1, ZDHHC1, ZDHHC2, and ZDHHC3 across the 32 TCGA cancer types. TIMER2.0 is implemented by Shiny for R (version 3.6.1) and generates a heatmap of Spearman’s correlation of gene expression (transcripts per million) between the PD-L1 and PD-1, DHHC1, DHHC2, and DHHC3, respectively. Additionally, purity adjustment was performed to obtain the purity-adjusted partial Spearman’s rho value of the tumor.

### Synthesis of cp-PCC degrader

The targeted DHHC3 binding peptide sequence for downregulating PD-L1 was derived from a computer-aided design of peptide sequences [43] and conjugated via solid-phase synthesis (Chinese Peptide Company, Hangzhou, China). The specific sequences and synthesis steps are shown in Supplementary Fig. S2. Peptides were conjugated at the N- and C-terminals with rhodamine and TAT (YGRKKRRQRRR) cell-penetrating peptides, respectively. The peptides were chemically synthesized and purified through high-performance liquid chromatography (HPLC) to achieve a purity exceeding 95%. This was followed by mass spectrometry (MS) for analysis. Additionally, three variants of the E3 ubiquitin ligase ligands were produced via chemical synthesis. These ligands were purified using HPLC to achieve a purity level above 98% and subsequently analyzed using MS (provided by the Chinese Peptide Company, Hangzhou, China). Similarly, cp-PCC was synthesized and purified to >95% purity using HPLC and its composition was confirmed using MS. Prior to application, cp-PCC was dissolved in sterile bi-distilled water and stored at −20 °C.

### Cell lines

Cell lines (C33A, MDA-MB-231, FaDu, A375, U14, 4T1, CT26, and B16F10) were provided by American Type Culture Collection (ATCC; Manassas, VA, USA) or its collaborators. All cell lines were cultured in a specific medium for general use (Supplementary Table S1). Human cervical cancer cell line C33A, mouse mammary carcinoma cell line 4T1, and mouse cervical cancer cell line U14 were purchased from ATCC. All cells were either recently purchased or authenticated by short tandem repeat profiling, authenticated and tested for mycoplasma contamination, and grown in 5% CO2 at 37 °C. All methods were performed in accordance with the relevant guidelines and regulations.

### Cell transfection

Small interfering RNA (siRNA) sequences were engineered to silence the expression of DHHC1, DHHC2 and DHHC3 in human tumor cell lines (Supplementary Table S1). C33A and MDA-MB231 cell lines in their logarithmic growth phase were selected for transfection. Following the protocol provided with the Lipofectamine 2000 reagent, diluted Lipofectamine™ 2000 was mixed with an equal volume of siRNA and incubated at room temperature for 10–15 min. The resulting liposome-siRNA complex was introduced into cells prepared for transfection. After 4–6 h, the medium was replaced with a complete culture medium. The cells were then incubated at 37 °C for 48–72 h. After incubation, the total cellular protein was harvested and changes in the expression of the target protein PD-L1 were assessed using Western Blot analysis.

### Western Blot assay

C33A, 4T1, and U14 cell lines were cultured in 6-well plates. These cells underwent treatment with compounds PCC16/17/18 in a series of concentrations (0, 0.1, 0.5, 5, 10, and 50 µM) for 4 h, and alternatively at a single concentration of 0.5 µM over 0, 2, 4, 8, 12, and 24 h. After treatment, the cells were collected and centrifuged at 1000 rpm for 5 min. The resulting cell lysates were prepared on ice using radioimmunoprecipitation assay (RIPA) buffer (Biosharp) containing protease inhibitors. This was followed by centrifugation at 13,000 × *g* for 30 min. The protein quantities were assessed using a bicinchoninic acid protein assay kit (Beyotime). The proteins were denatured by heating at 100 °C for 10 min before electrophoresis. Sodium dodecyl-sulfate polyacrylamide gel electrophoresis gels of a suitable concentration were prepared, and 30 µg of protein from each sample was loaded for electrophoresis, followed by transfer onto poly vinylidene fluoride (PVDF) membranes. The membranes were then blocked with 5% non-fat milk at room temperature for 2 h. Primary antibody incubation was performed overnight at 4 °C. This was followed by three 10-min washes in TBST, incubation with secondary antibodies at room temperature for 1 h, and another series of three 10-min tris-buffered saline with 0.1% Tween 20 detergent (TBST) washes. For protein detection, the PVDF membranes were treated with a 1:1 solution of an enhanced chemiluminescence reagent (Vazyme) and subsequently visualized using a ChemiDoc XRS system (Bio-Rad Laboratories). This allowed the recording of protein expression levels as a part of the experimental data.

### Flow cytometric analysis

C33A cells, post-incubation with PCC16/17/18 drugs at concentration gradients of 0, 0.1, 0.5, 5, 10, and 50 µM for 4 h or at a concentration of 0.5 µM for 0, 2, 4, 8, 12, and 24 h, were digested with 0.05% trypsin and centrifuged at 1000 rpm for 5 min. The supernatant was discarded, the cell pellet was collected, which was washed twice with ice-cold phosphate buffered saline (PBS), and the cell fluorescence intensity was measured using the PE-A channel of a flow cytometer, ensuring that light exposure was minimized. A total of 10,000 cells were used in each experiment.

### Cell thermal shift assay

C33A cells were seeded in a 6-well plate, trypsinized, and washed with PBS. The cells were then resuspended in PBS containing protease inhibitors. Aliquots of 500 µL (control and 1 µM PCC16 treated) in 1.5 mL reaction tubes were heated separately at 37, 40, 43, 46, 49, 52, 55, 58, and 61 °C for 3 min each, followed by cooling at room temperature for 3 min, and flash freezing in liquid nitrogen and storing at −80 °C. For each thermal denaturation point, the frozen cell samples were lysed in RIPA buffer containing protease inhibitors, incubated on ice for 15 min, and sonicated for 10 s at 4 °C. The lysates were centrifuged at 20,000 × *g* for 20 min. The supernatants were transferred to new tubes and prepared for Western Blot analysis. Western Blot analysis was performed and the bands were quantified using the aforementioned method.

### Immunofluorescence staining

PCC16/17/18 cells were labeled with rhodamine to observe their intracellular distribution and interactions with target proteins using immunofluorescence. After treatment with 5 µM of PCC16/17/18 for 4 h, the culture medium was removed, and cells were washed with PBS. The cells were then fixed in 4% paraformaldehyde in a 6-well plate for 30 min at room temperature to ensure protection from light. Subsequently, cells were permeabilized with 0.3% Triton X-100 in PBS for 15 min at room temperature. After additional permeabilization with 0.5% Triton for 15 min and two washes with PBS for 5 min each, the cells were blocked with 1% bovine serum albumin for 30–60 min. Primary antibodies diluted in an antibody diluent targeting DHHC3 or PD-L1 were added and incubated overnight at 4 °C in a humidified chamber. After three washes with PBS with Tween20 (PBST), each lasting 5 min, cells were incubated with 50 µL of a specific secondary antibody conjugated to a fluorescent marker, diluted in antibody diluent for 1 h at room temperature in the dark. Following three more PBST washes, cells were stained with 4’,6-diamidino-2-phenylindole (DAPI) for 3–5 min and washed three times with PBS. A drop of antifade mounting medium was applied to a microscope slide, and the cells were mounted face-down on the slide for observation under a fluorescence microscope. Imaging was performed using a Nikon Eclipse Ni-U microscope equipped with a ProgRes MFcool camera. Digital images were processed using ImageJ software (NIH).

### T cell–cancer cell co-culture

Peripheral blood was drawn from a healthy individual in their 20 s for isolation of human peripheral blood mononuclear cells. These cells were separated using a lymphocyte separation medium via the density gradient method. CD3^+^ T cells were isolated and concentrated using an Easy Sep Human T Cell Isolation Kit. The T cells were then adjusted to a density of 1 × 10^6^ cells/mL. For co-culture, the T cells were incubated with anti-CD3 (1 µg/mL), anti-CD28 (1 µg/mL), and IL-2 (10 ng/mL) for 2 days. This process was augmented with additional anti-CD3/28 and IL-2 for another 2–3 days to expand the T cell population for the upcoming phases of the experiment. Concurrently, cancer cells, pre-treated with 10 µg/mL of mitomycin, were resuspended in the culture medium and combined with the T cells in a 96-well plate at a predefined cell ratio. Following exposure to varying concentrations of PCC16 (0, 1, 5, 10 µM), the 96-well plate underwent a PBS wash, and the cells were stained using Hoechst 33258 (Biosharp). After two additional PBS washes, cells were examined and imaged under a fluorescence microscope.

### Enzyme-linked immunoassay

To establish the described T cell-cancer cell co-culture system, PCC16 was introduced in a concentration gradient of 0, 0.1, 0.5, 5, 10, and 50 µM and incubated for 4 h. Then, the cultures were centrifuged at 350 × *g* for 5 min at 4 °C, and the supernatant from the cell culture was collected. The concentrations of cytokines IFN-γ and TNF-α were determined using specific enzyme-linked immunoassay (ELISA) kits (Nebioscience, China) as per the provided instructions. An ELISA reader was used to measure the optical density values for each group to estimate the cytokine concentrations. These procedures were repeated thrice to ensure accuracy and reliability.

### MTT assay

Cells were evenly distributed in a 96-well plate, with each well containing 3000–5000 cells. This setup was replicated thrice under each experimental condition. A total of 100 µL of culture medium was added to every well, and the outer wells were filled with an appropriate amount of PBS to minimize medium evaporation. Control wells containing only 100 µL of the medium without any cells were also prepared. The plate was then placed in a cell culture incubator and replicated three times (*n* = 3). Upon reaching nearly 80% confluence, the cells were prepared for drug treatment, the duration of which varied depending on the specificity of the experiment. After removing the culture medium, 20 µL of the MTT reagent was administered to each well, and the plate was returned to the incubator for 4 h. After incubation, the MTT reagent was removed, and 150 µL of dimethyl sulfoxide was added to dissolve the formazan crystals. The plate was gently agitated on a shaker for 5 min to ensure proper mixing. The absorbance of each well was measured using an ELISA plate reader. To analyze the data, a graph was plotted with the drug concentration on the x-axis and cell viability on the y-axis. Cell viability was computed using the following formula: [(Absorbance of the experimental well − Absorbance of the blank well)/(Absorbance of the control well − Absorbance of the blank well)].

### Terminal deoxynucleotidyl transferase dUTP nick end labeling assay

Cells were seeded in 6-well plates at a density between 30% and 50%. Following complete adhesion, various treatments were applied: PBS alone, cisplatin, or a combination of cisplatin and PCC16 for 4 h each. After incubation, the medium was replaced and the cells were rinsed with PBS. The cells were fixed with 4% paraformaldehyde for half an hour and washed again with PBS. To permeabilize the cells, PBS mixed with 0.3% Triton X-100 was added and the cells were incubated at room temperature for 5 min. A specific quantity of the terminal deoxynucleotidyl transferase dUTP nick end labeling (TUNEL) assay reagent was prepared and mixed. After washing the cells on coverslips with PBS twice, 50 µL of the TUNEL reagent was applied to each cell sample. These samples were then incubated at 37 °C in a dark environment for 60 min. The samples were then washed three times with PBS and stained with DAPI for 3–5 min, followed by washing with PBS twice. The final step involved mounting the samples for observation under a fluorescence microscope using an excitation wavelength range of 450–500 nm and an emission wavelength range of 515–565 nm to detect green fluorescence.

### Colony formation assay

The procedure commenced by counting and seeding cancer cells into a 6-well plate, with each well containing approximately 1000 cells. Following complete cell adhesion, various treatments were applied to each well, including a control PBS solution, cisplatin alone, and a combination of cisplatin and PCC16. This was followed by a 4-h incubation period. Subsequently, the initial culture medium was discarded, and the cells were incubated for additional 7 days in a fresh complete medium, which was renewed every 2–3 days. The cultivation process was halted on the formation of visible colonies detectable under a microscope, with each colony comprising approximately 60–80 cells. Double washing with PBS was conducted, followed by a 20-min fixation of the cells using a tissue cell fixative. After fixation, the cells were washed twice with PBS and stained with 0.1% crystal violet for 20 min. After rinsing the excess stain with deionized water, the plates were air-dried at ambient temperature. The colonies were photographed for documentation, and the cell colony counts in each treatment group was recorded.

### Experimental mouse models and therapeutic procedures

Female BALB/c mice, aged 6–8 weeks and weighing approximately 20 g were procured from the Wuhan Experimental Animal Center, Hubei, China. In vivo studies assessing antitumor effects were performed according to the protocols of the Experimental Animal Welfare and Ethics Committee at Wuhan University. These studies complied with the National Research Council’s guidelines detailed in the “Guide for the Care and Use of Laboratory Animals.” Cells were harvested in an optimal growth phase with approximately 80% confluence, centrifuged to discard the supernatant, and 2 million 4T1 cells were resuspended in 125 µL of PBS. This cell suspension was subcutaneously injected into the dorsal flank of mice to initiate the tumor model. Tumor dimensions were regularly measured using calipers, and the volume was calculated using the following formula: (length × width²)/2. When the tumor volume was 80–100 mm³, the tumor-bearing mice were allocated for further in vivo investigation. The mice were grouped into four categories (each containing four mice): a control group, a group receiving anti-PD-L1 monoclonal antibody, a group treated with the PD-L1 inhibitor BMS-8, and a group administered PCC16. The treatments were administered via tail vein injections on days 0, 2, 4, 6, 8, 10, and 12. Throughout the experiment, both the weight of the mice and tumor volume were recorded. On day 18, the mice were euthanized via cervical dislocation. The tumors were excised for measurement, photographic documentation, and weighing. One segment of each tumor was set aside for Western Blot analysis, and another was fixed in 4% paraformaldehyde and embedded in paraffin for further examination. The major organs, including the heart, liver, spleen, lungs, and kidneys, were extracted, fixed, and embedded in paraffin. Subsequent histological examinations of the tissues were conducted after staining with hematoxylin and eosin and observing under a microscope. All methods were performed in accordance with the relevant guidelines and regulations.

### Immunohistochemistry

Immunohistochemistry was performed according to manufacturer’s instructions provided with the kit. Following deparaffinization and rehydration, the sections were immersed in EDTA buffer solution for high-temperature antigen retrieval. Subsequently, 3% H2O2 was added to block endogenous peroxidase activity. PD-L1 antibody concentration was prepared according to the antibody’s datasheet, and the sections were incubated overnight at 4 °C (for over 8 h). The next day, the sections were brought to room temperature in a 37 °C incubator for 1 h, followed by the addition of an enhancer solution and incubation for 20 min. The secondary antibody was added and incubated for 20 min. The 3,3′-Diaminobenzidine chromogen was applied to the samples, followed by counterstaining with hematoxylin. Sections were dehydrated using graded alcohol and mounted with a neutral resin.

### Statistical methodology

Data visualization and statistical evaluation were performed using GraphPad Prism software version 8.1.1. Each experiment was conducted in triplicate, and the results are presented as the mean ± standard error of the mean. For comparisons between two distinct groups, a two-tailed t-test was used. In cases involving comparisons across more than two groups, one-way analysis of variance was employed, followed by specific post hoc analyses (either Tukey’s or Dunnett’s tests), as applicable. Statistical significance was set at *p* < 0.05. Significance levels are indicated as follows: *P < 0.05, **P < 0.01, and ***P < 0.001.

## Supplementary information

Supplemental material

Source data of Werstern Blot

## Data Availability

All data needed to evaluate the conclusions in this study are present in this article and Supplementary Materials. Additional data related to this paper may be requested from the authors.
