# Correction: Treating ICB-resistant cancer by inhibiting PD-L1 via DHHC3 degradation induced by cell penetrating peptide-induced chimera conjugates

**DOI:** 10.1038/s41419-024-07182-8

**Published:** 2024-11-18

**Authors:** Yu-Ying Shi, Gang Fan, Ruirong Tan, Shan Li, Hua-Bing Sun, Rui Li, Mengni Yang, Shanshan Gao, Miao Liu, Meng-Yuan Dai

**Affiliations:** 1 Department of Gynecological Oncology, Zhongnan Hospital of Wuhan University, Wuhan, China; 2 Department of Urology, Huazhong University of Science and Technology Union Shenzhen Hospital, Shenzhen, China; 3 China Translational Chinese Medicine Key Laboratory of Sichuan Province, State Key Laboratory of Quality Evaluation of Traditional Chinese Medicine, Sichuan Institute for Translational Chinese Medicine, Sichuan Academy of Chinese Medicine Sciences, Chengdu, China; 4 Tianjin Key Laboratory on Technologies Enabling Development of Clinical Therapeutics and Diagnostics, School of Pharmacy, Department of Nuclear Medicine, Tianjin Medical University General Hospital, Tianjin Medical University, Tianjin, China; 5 Department of Radiation Oncology, Radiation Oncology Key Laboratory of Sichuan Province, Sichuan Clinical Research Center for Cancer, Sichuan Cancer Hospital and Institute, Sichuan Cancer Center, Chengdu, China; 6 Department of Biomedical Sciences, Faculty of Health Sciences, University of Macau, Taipa, Macau, China; 7 Department of Pathology, Brigham and Women’s Hospital, Harvard Medical School, Boston, MA USA

**Keywords:** Immune cell death, Tumour biomarkers

Correction to: *Cell Death and Disease* 10.1038/s41419-024-07073-y, published online 30 September 2024

In the original version of this article, Fig. 4A does not show the correct resolution of confocal images to illustrate the binding of DHHC3 target with the drug PCC16. The corrected Fig. 4A is provided below. This unintentional mistake does not affect the conclusions of the study. The authors apologize for any inconvenience this may have caused.

Amended Fig. 4
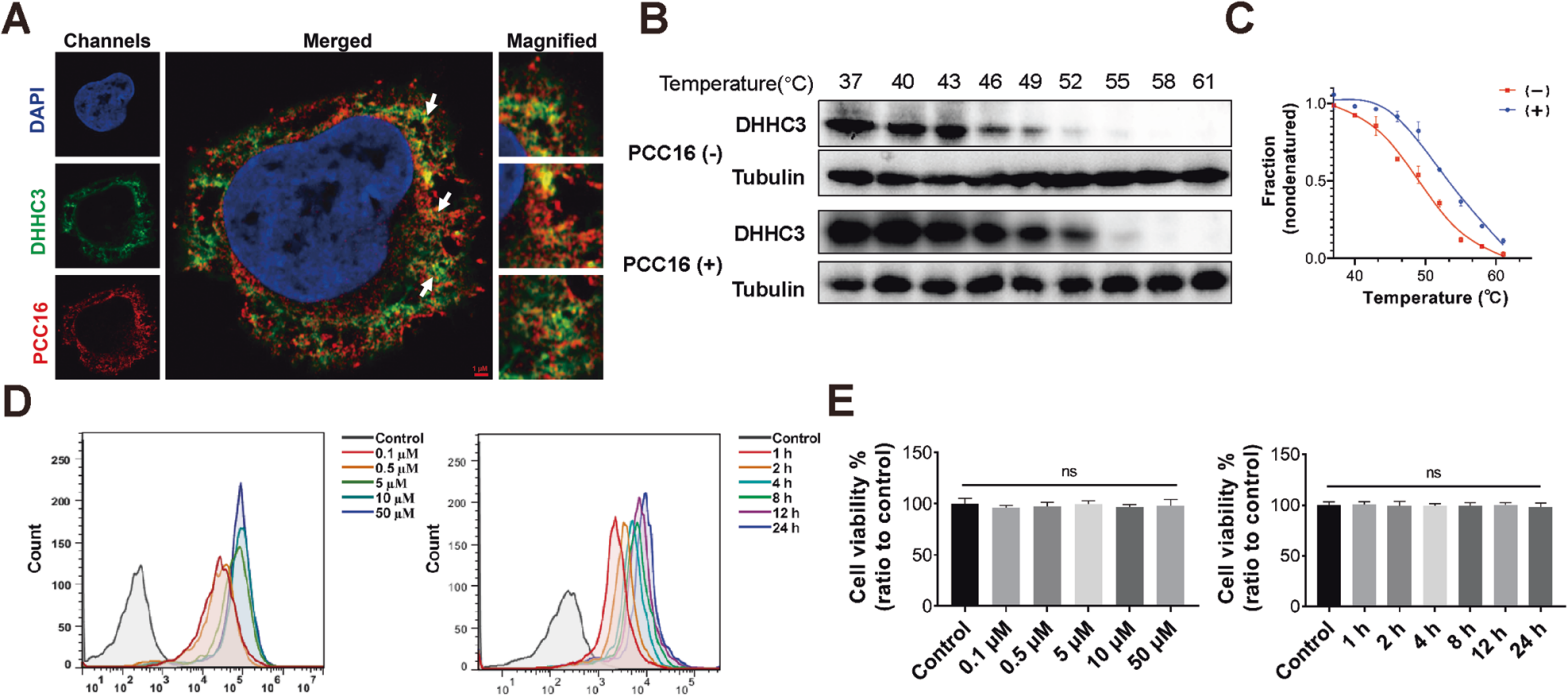


The original article has also been corrected.

