# Supplementary material for: Treating ICB-resistant cancer by inhibiting PD-L1 via DHHC3 degradation induced by cell penetrating peptide-induced chimera conjugates
Source: Cell Death Dis. 2024 Sep 30;15(9):701. doi: 10.1038/s41419-024-07073-y (PMC11442653; doi:10.1038/s41419-024-07073-y)
Supplement: Supplementary file 1 — Supplemental material [file 41419_2024_7073_MOESM1_ESM.pdf]

Supporting Information for

# Treating ICB-Resistant Cancer by Inhibiting PD-L1 via DHHC3 Degradation Induced by Cell Penetrating Peptide-Induced Chimera Conjugates

Yu-Ying Shi [a#], Ruirong Tan[b#], Gang Fan[c#], Shan Li [a], Hua-Bing Sun[d], Rui Li[e], Mengni Yang[b], Shanshan Gao[f], Miao Liu[g *], Meng-Yuan Dai[a*]

*Corresponding author(s)：mengyuandai@whu.edu.cn (Meng-Yuan Dai), mliu0@bwh.harvard.edu (Miao Liu).

**The PDF file includes：**

Table S1

Figs. S1 to S4

# Table of Contents

1. Table S1 Cells、siRNAs and REAGENTS…………………………………………..…3

2. FigS1 PD-L1 protein levels across all cell types with treatment of PCC16……………..……4

3. FigS2 The structures and synthetic routes of PCC17/18 ………………………….……..….4

4. FigS3 Quality Control Analysis for Cyclic Peptide for PCC17 / 18 …..……………..……6

5. FigS4 PD-L1 protein levels in 4T1 cells treated with PCC16………..……….……….…...10

## 1. Table S1

| CELL LINES | SOURCE | CULTURE CONDITION |
|---|---|---|
| C33A | Human cervical cancer cell | MEM+10%FBS+1%P/S |
| MDA-MB-231 | Human breast cancer cell | Leibovitz's L-15+10%FBS +1% P/S |
| FaDu | Human squamous cell carcinoma cell | RPMI-1640+10%FBS+1%P/S |
| A375 | Human melanoma cell | DMEM+10%FBS+1%P/S |
| U14 | Mouse cervical cancer cell | DMEM+10%FBS+1%P/S |
| 4T1 | Mouse breast cancer cell | RPMI-1640+10%FBS+1%P/S |
| SCC7 | Mouse squamous cell carcinoma cell | DMEM +10%FBS+1%P/S |
| CT26 | Mouse colon cancer cell | RPMI-1640+10%FBS+1%P/S |
| B16F10 | Mouse melanoma cell | RPMI-1640+10%FBS+1%P/S |
| siRNA Name | Sense | Antisense |
| si-DHHC1 | GCACGCACAUGUCAUUGAATT | UUCAAUGACAUGUGCGUGCTT |
| si-DHHC2 | AAGGAUCUUCCCAUCUAUUTT | AAUAGAUGGGAAGAUCCUUGG |
| si-DHHC3 | CCUCAAAGUGGAUUCACUUTT | AAGUGAAUCCACUUUGAGGTT |
| REAGENTS | SOURCE | IDENTIFIER |
| Anti-PDL1 antibody | Abcam | ab205921; ab213480 |
| Anti-GDOZ/DHHC3 antibody | Abcam | ab31817 |
| Anti-GAPDH | Abcam | ab9485 |
| Anti-Alpha Tubulin | Cell signaling technology | Cat#2144S |
| Goat anti-mouse secondary antibody | Proteintech | SA00001-1 |
| Goat anti-rabbit secondary antibody | Proteintech | SA00001-2 |
| MG132 | Sigma | M7449 |
| Cisplatin | MCE | HY-17394 |
| Lipofectamine 2000 | Invitrogen | 11668-030 |
| Anti-Human CD3 | BD Pharmingen™ | 555329 |
| Anti-Human CD28 | BD Pharmingen™ | 555725 |
| Recombinant Human IL-2 | BD Pharmingen™ | 554603 |
| RecombiMAb anti-mouse PD-L1 | Bio X Cell | #CP168 |
| BMS-8 | MCE | HY-116274 |

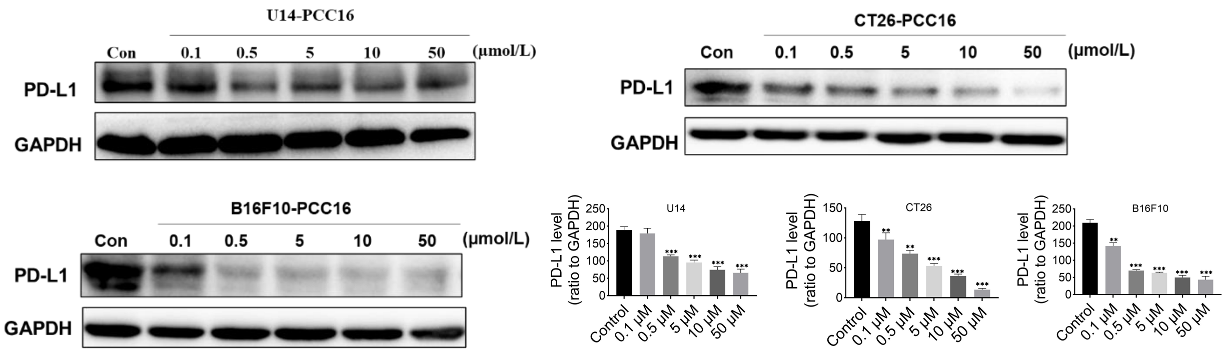


**Fig S1:** Expression of PD-L1 protein in U14/CT26/B16F10 cell lines after 6h treatment with different concentrations of PCC16; Quantitative data expressed as mean ± standard error; analyzed by one ANOVA with Tukey's post hoc test (n = 3): *P < 0.05, **P < 0.01, ***P < 0.001.

Peptide：PCC17

Mol Formula：$C_{215}H_{341}N_{68}O_{59}S_2^{+}$

Mol Weight:  4886.56±1.0

Structure:

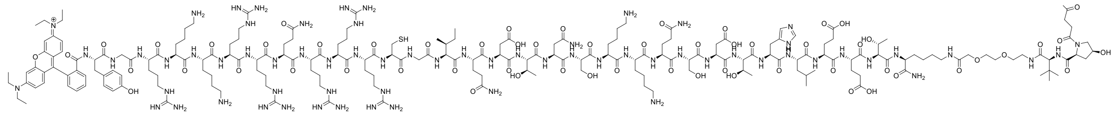

Synthetic Route:

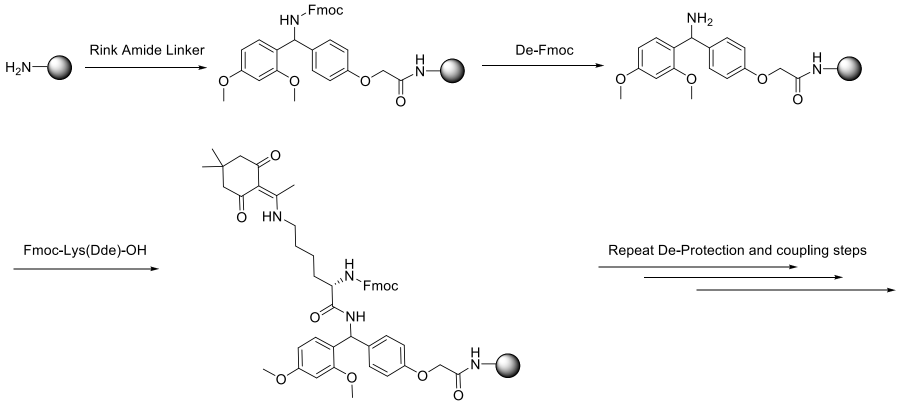


RhodmineB-Tyr(tBu)-Gly-Arg(pbf)-Lys(Boc)-Lys(Boc)-Arg(pbf)-Arg(pbf)-Gln(Trt)-Arg(pbf)-Arg(pbf)-Arg(pbf)-Cys(Trt)-Gly-Ile-Gln(Trt)-Asp(OtBu)-Thr(tBu)-Asn(Trt)-Ser(tBu)-Lys(Boc)-Lys(Boc)-Gln(Trt)-Ser(tBu)-Asp(OtBu)-Thr(tBu)-His(Trt)-Leu-Glu(OtBu)-Glu(OtBu)-Thr(tBu)-Lys([4-(4-methyl-1,3-thiazol-5-yl)phenyl]methanamine-Suc-Hyp(tBu)-Tle-PEG2)-Rink Amide MBHA Resin

Cleavage & Purification

RhodmineB-Tyr-Gly-Arg-Lys-Lys-Arg-Arg-Gln-Arg-Arg-Arg-Cys-Gly-Ile-Gln-Asp-Thr-Asn-Ser-Lys-Lys-Gln-Ser-Asp-Thr-His-Leu-Glu-Glu-Thr-Lys([4-(4-methyl-1,3-thiazol-5-yl)phenyl]methanamine-Suc-Hyp-Tle-PEG2)-NH2

Custom Peptide

Peptide：PCC18

Mol Formula：$C_{224}H_{355}N_{68}O_{59}S^{+}$

Mol Weight: 4976.70±1.0

Structure:

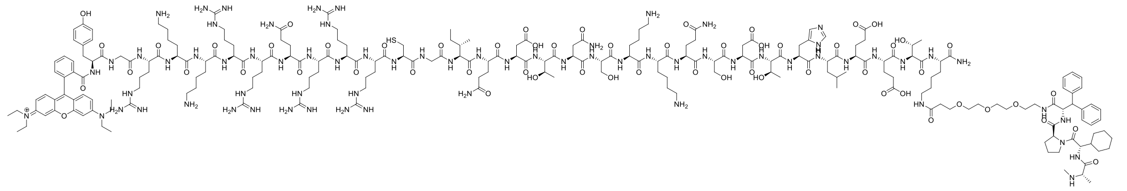

Synthetic Route:

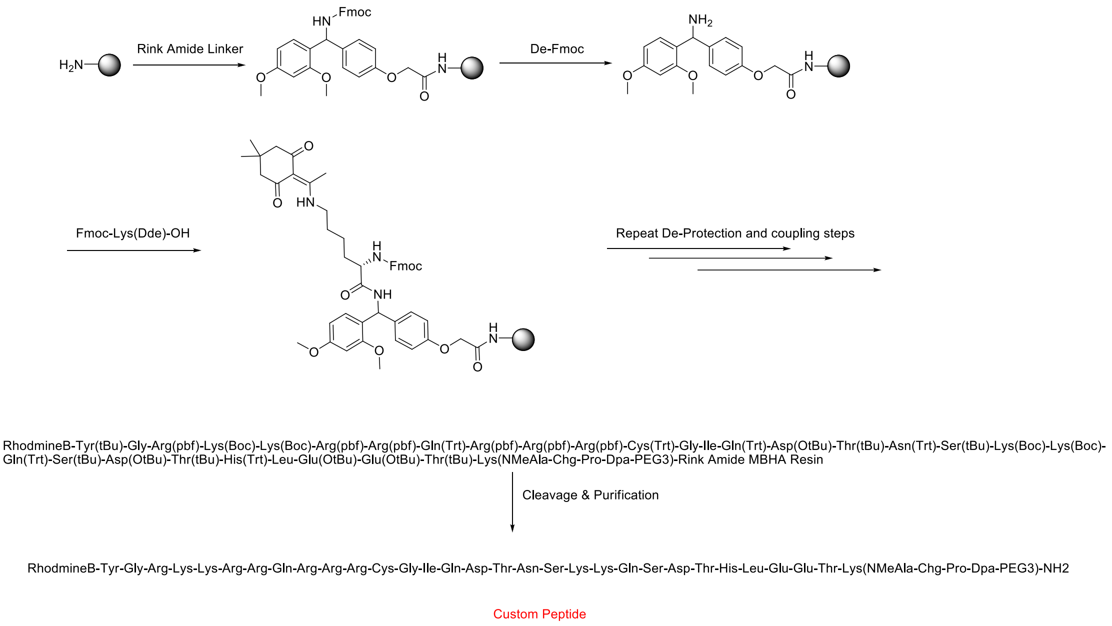


**Fig S2:** The structures and synthetic routes of PCC17/18

**Fig S3  Quality Control Analysis for Cyclic Peptide for PCC17/18**

```
Sample Name  PCC17
Mobile Phase:A:0.1%TFA in H2O
             B:0.09%TFA in (80%ACN+20%H2O)
Flow:1.0ml/min  33.0%-43.0%  B buffer in 20min
Column:SepaxGP-C18 5u 120A 4.6*150mm A1230# 60C
=========================================================================>
                                              Location    :      Vial 7
                                              Inj. Vol.   :       40 µl
```

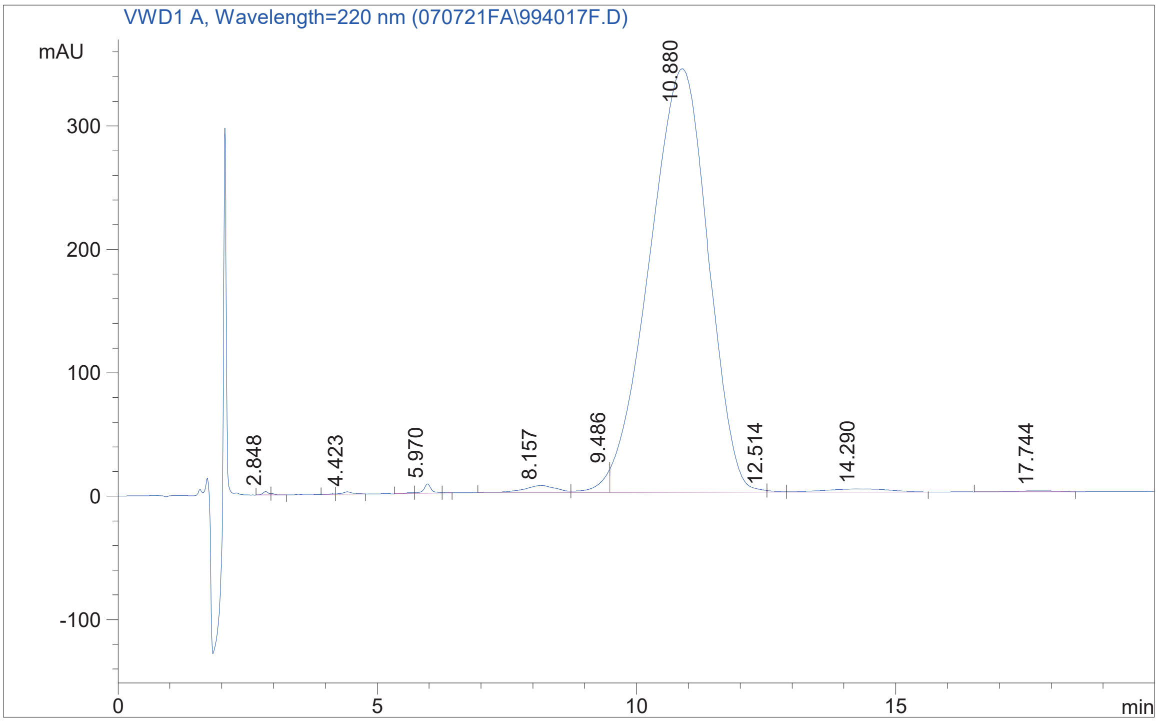


===========================================================================>

Signal  1:VWD1 A, Wavelength=220 nm

| Peak # | RT [min] | Type | Height | Width [min] | Area | Area % |
|---|---|---|---|---|---|---|
| 1 | 2.848 | BF | 2.805 | 0.115 | 21.393 | 0.077 |
| 2 | 2.953 | VP | 1.031 | 0.098 | 6.041 | 0.022 |
| 3 | 4.147 | PV | 0.452 | 0.112 | 3.449 | 0.012 |
| 4 | 4.423 | VBA | 1.853 | 0.188 | 24.383 | 0.088 |
| 5 | 5.628 | PV | 0.716 | 0.157 | 7.503 | 0.027 |
| 6 | 5.970 | VV | 7.536 | 0.146 | 73.362 | 0.265 |
| 7 | 6.337 | VV | 0.290 | 0.116 | 2.227 | 0.008 |
| 8 | 8.157 | BV | 5.535 | 0.598 | 231.358 | 0.835 |
| 9 | 9.486 | VV | 18.911 | 0.202 | 229.688 | 0.829 |
| 10 | 10.880 | VF | 343.122 | 1.218 | 26823.807 | 96.840 |
| 11 | 12.514 | VV | 1.328 | 0.184 | 14.658 | 0.053 |
| 12 | 14.290 | VV | 2.511 | 1.070 | 217.152 | 0.784 |
| 13 | 17.744 | VP | 0.831 | 0.655 | 44.133 | 0.159 |

============================================================================

PCC17:
MW:4886.6

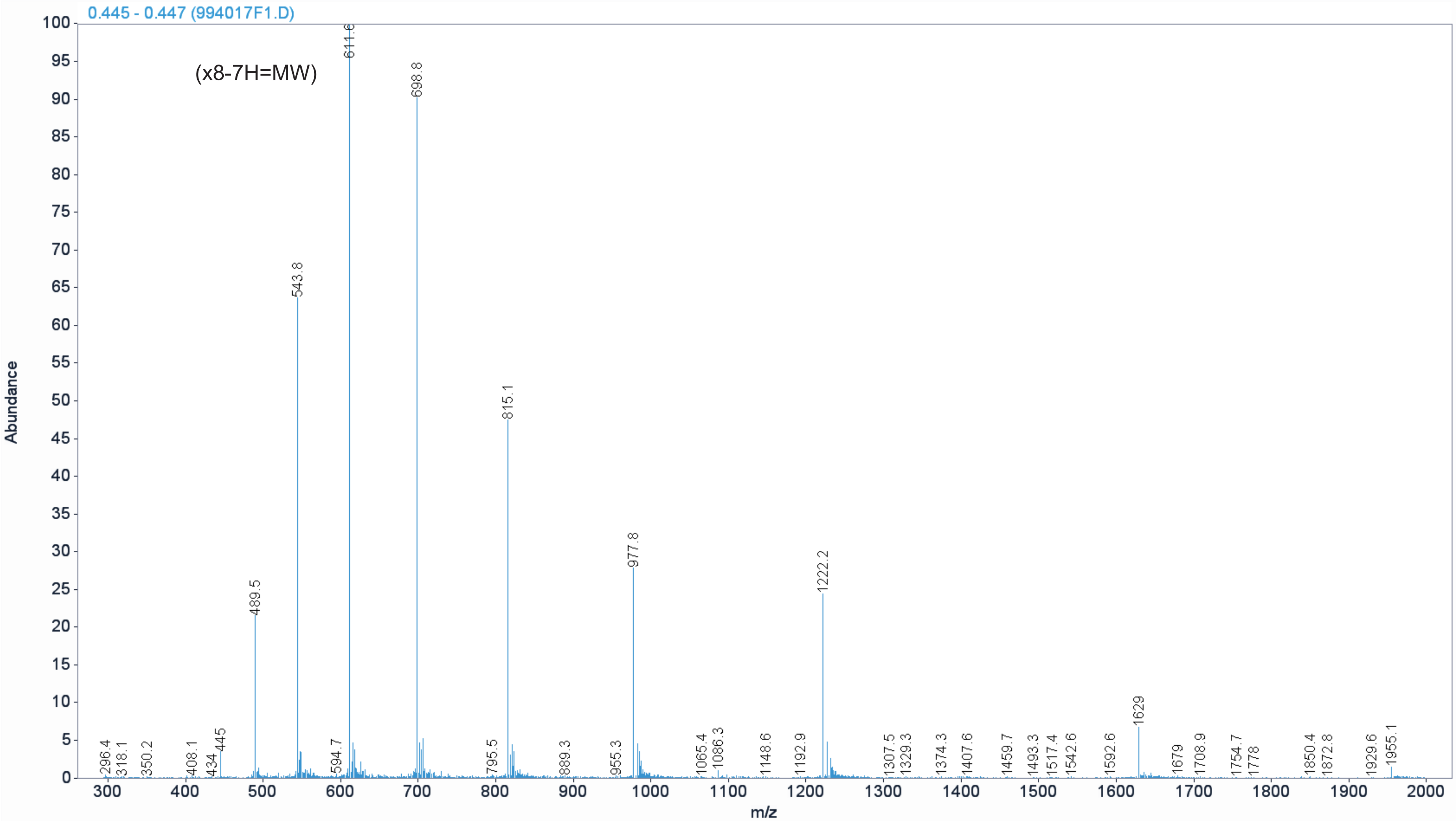

Sample Name:PCC18
Mobile Phase:A:0.1%TFA in H2O
B:0.09%TFA in (80%ACN+20%H2O)
Flow:1.0ml/min 37.0%-47.0% B buffer in 20min
Column:SepaxGP-C18 5u 120A 4.6*150mm A1230# 60C

================================================================================>

Location : Vial 1
Inj. Vol. : 40 µl

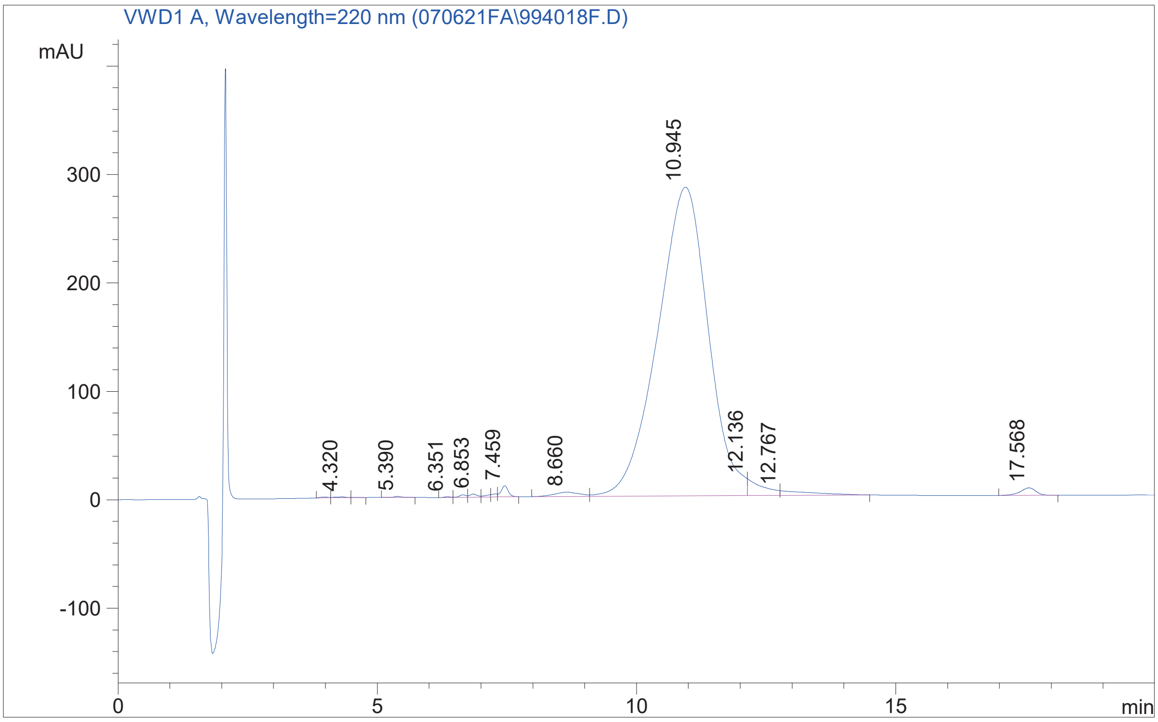


================================================================================>

Signal 1:VWD1 A, Wavelength=220 nm

| Peak # | RT [min] | Type | Height | Width [min] | Area | Area % |
|---|---|---|---|---|---|---|
| 1 | 3.972 | BV | 0.765 | 0.165 | 7.973 | 0.039 |
| 2 | 4.320 | VV | 0.794 | 0.204 | 11.703 | 0.058 |
| 3 | 4.609 | VV | 0.340 | 0.171 | 3.534 | 0.017 |
| 4 | 5.390 | BBA | 0.915 | 0.183 | 11.177 | 0.055 |
| 5 | 6.351 | BV | 0.724 | 0.127 | 5.915 | 0.029 |
| 6 | 6.653 | VV | 2.295 | 0.135 | 20.776 | 0.103 |
| 7 | 6.853 | VV | 2.856 | 0.151 | 29.216 | 0.144 |
| 8 | 7.185 | VV | 2.000 | 0.134 | 16.085 | 0.079 |
| 9 | 7.275 | VV | 2.720 | 0.110 | 20.269 | 0.100 |
| 10 | 7.459 | VBA | 10.188 | 0.145 | 96.127 | 0.475 |
| 11 | 8.660 | BV | 4.022 | 0.475 | 134.559 | 0.665 |
| 12 | 10.945 | VF | 284.652 | 1.036 | 19232.287 | 95.030 |
| 13 | 12.136 | VF | 15.576 | 0.342 | 319.333 | 1.578 |
| 14 | 12.767 | VBA | 4.366 | 0.700 | 183.296 | 0.906 |
| 15 | 17.568 | BBA | 6.961 | 0.317 | 145.817 | 0.721 |

================================================================================

*** End of Report ***

PCC18
MW:4962.7

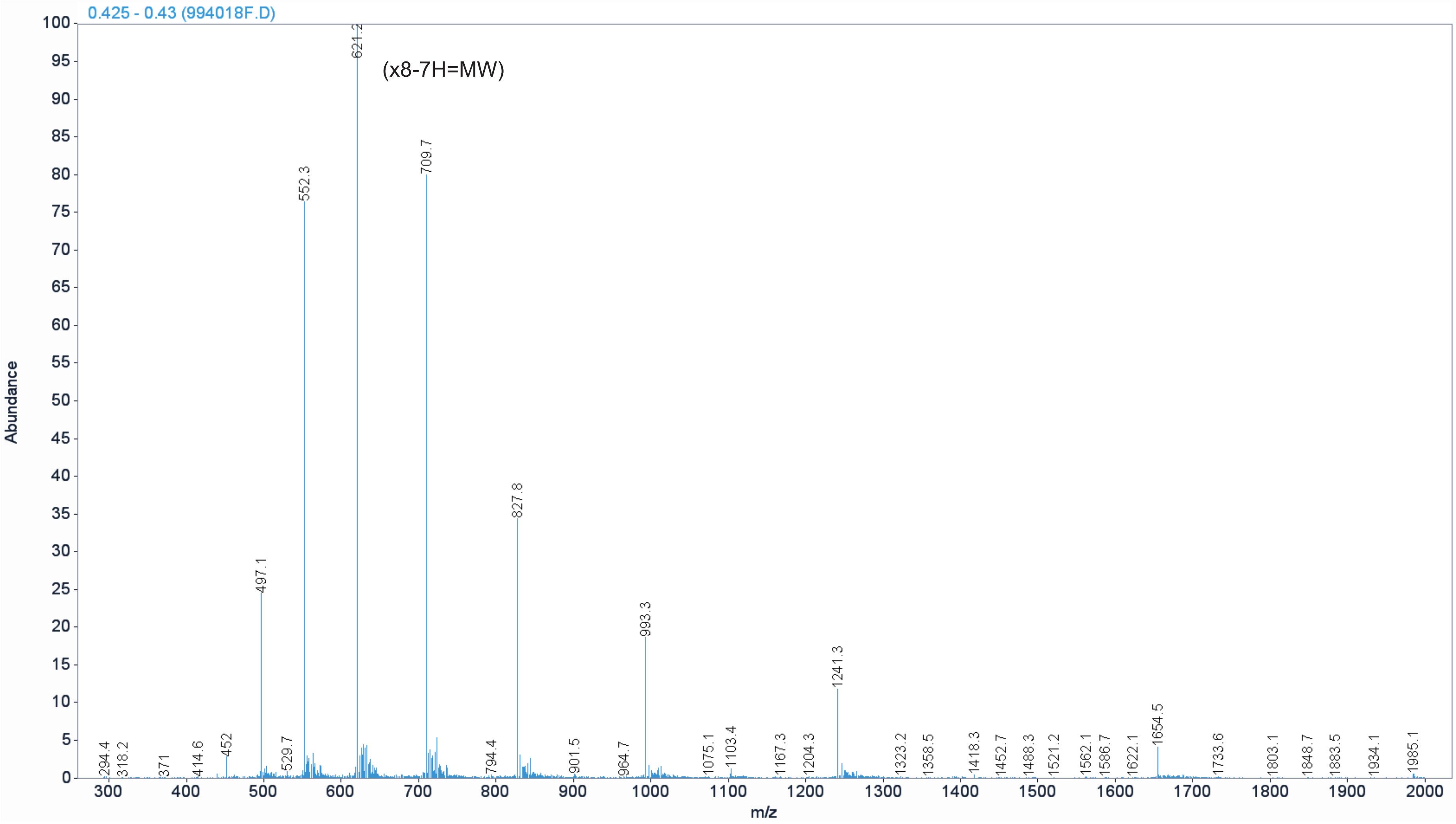

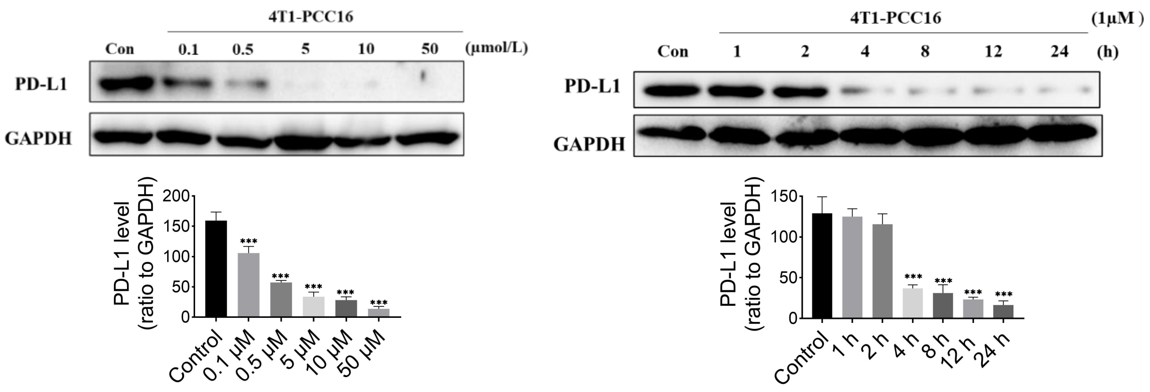


**Fig S4:** A. Western blot analysis of PD-L1 protein expression in 4T1 cells treated with various concentrations of PCC16 for 6 hours; B. Western blot analysis of PD-L1 protein expression in 4T1 cells treated with 1μM PCC16 over different time intervals;
