# Supplementary figures and images for: Treating ICB-resistant cancer by inhibiting PD-L1 via DHHC3 degradation induced by cell penetrating peptide-induced chimera conjugates
Source: Cell Death Dis. 2024 Sep 30;15(9):701. doi: 10.1038/s41419-024-07073-y (PMC11442653; doi:10.1038/s41419-024-07073-y)

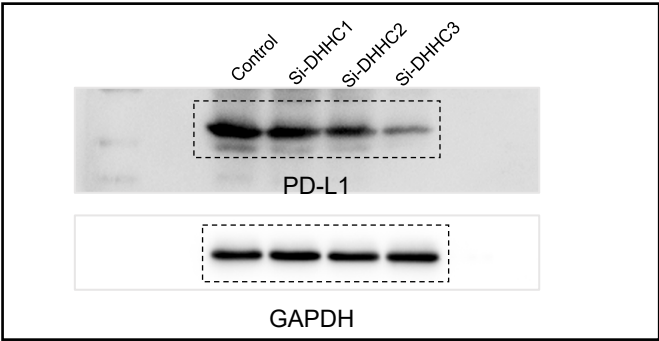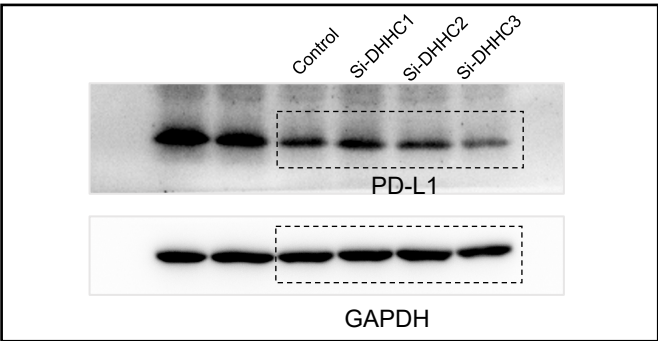

Figure 1c

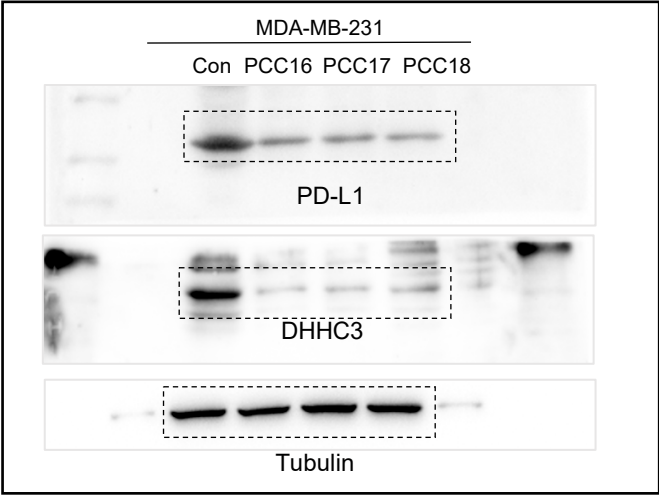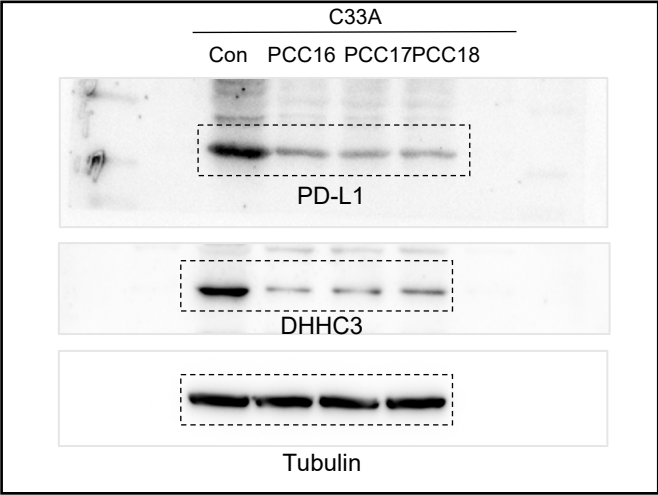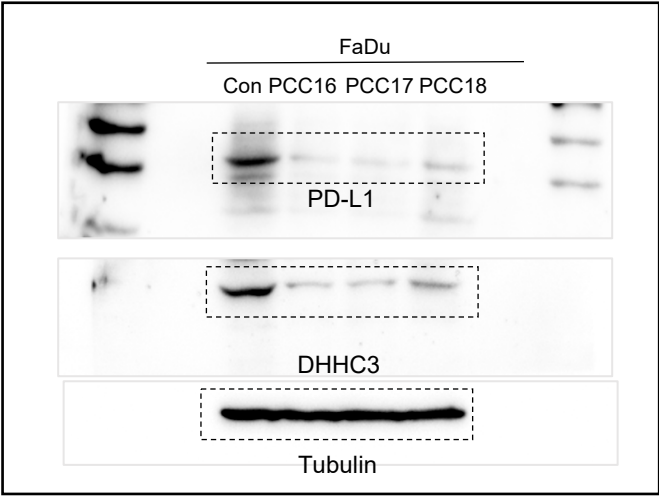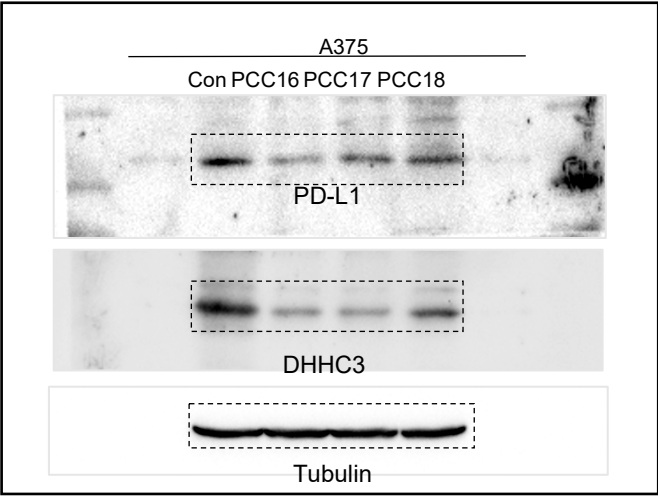

Figure 1e

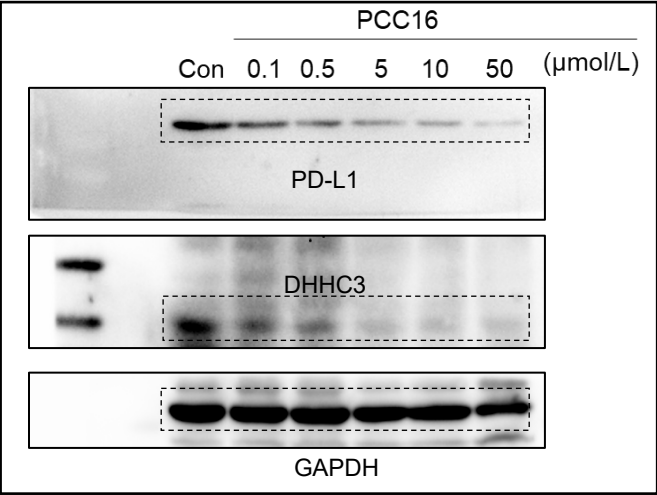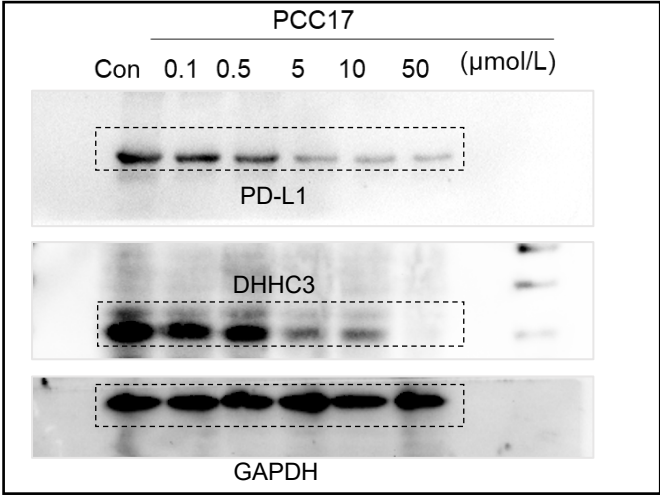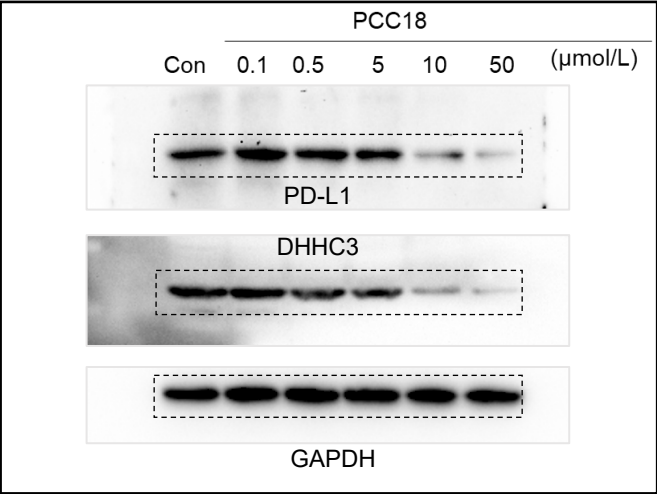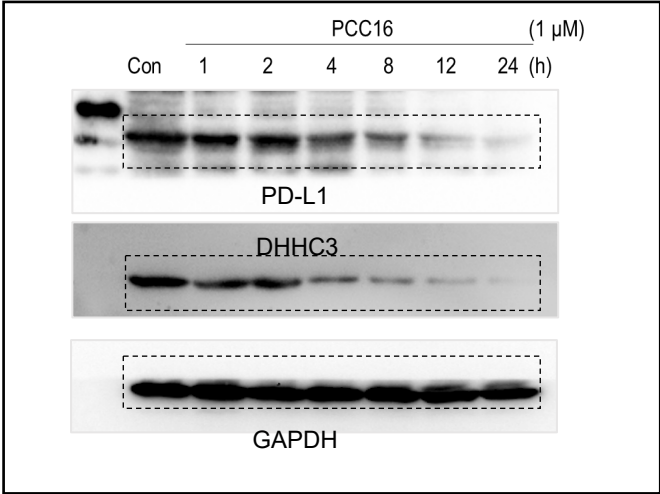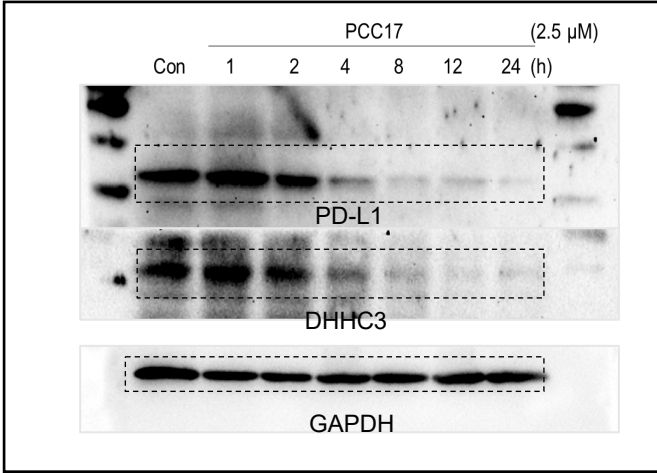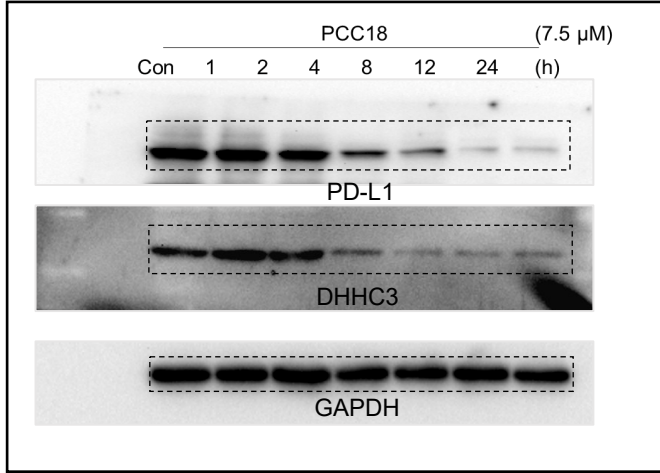

Figure 2

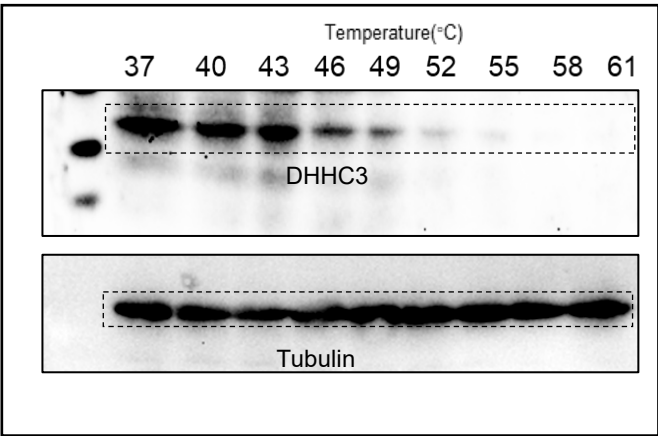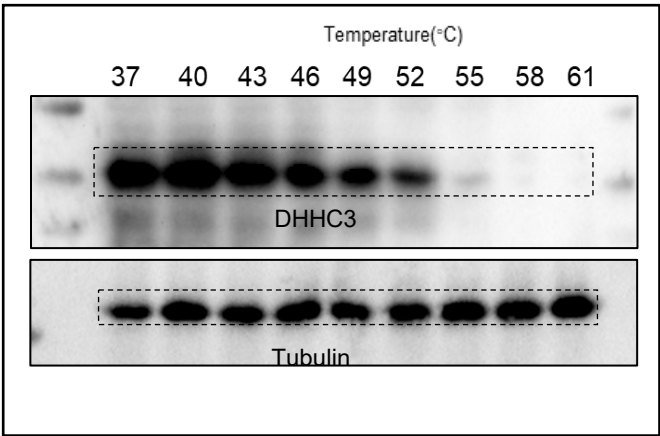

Figure 4b

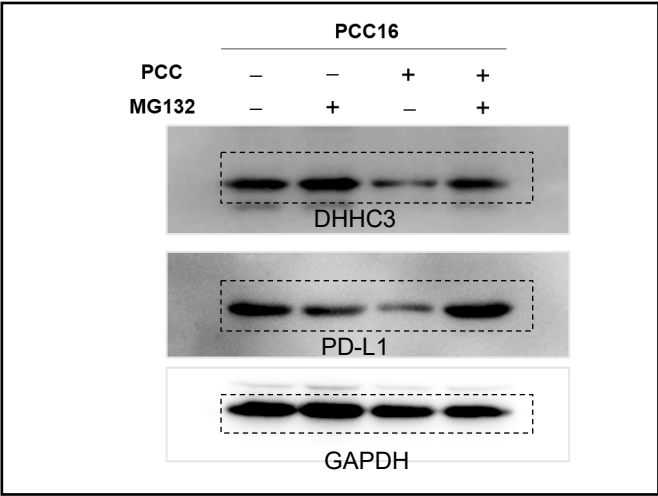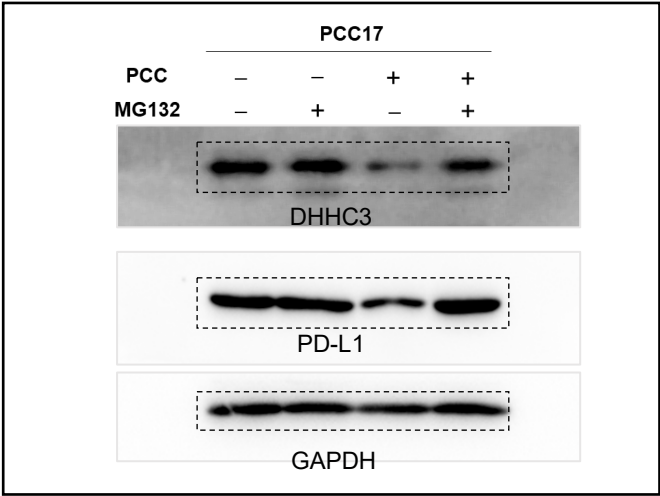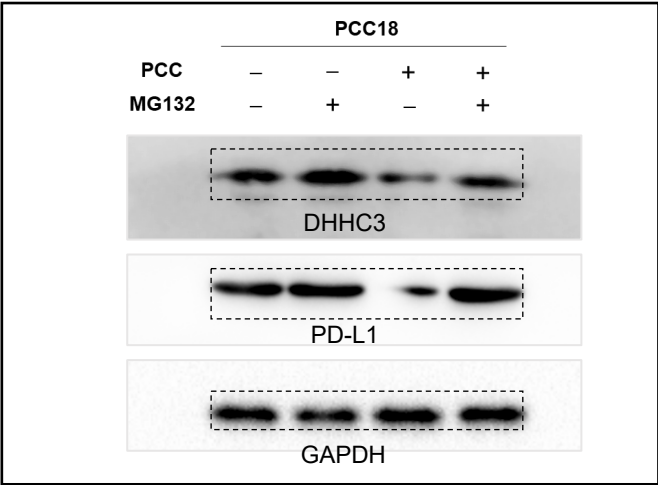

Figure 5a

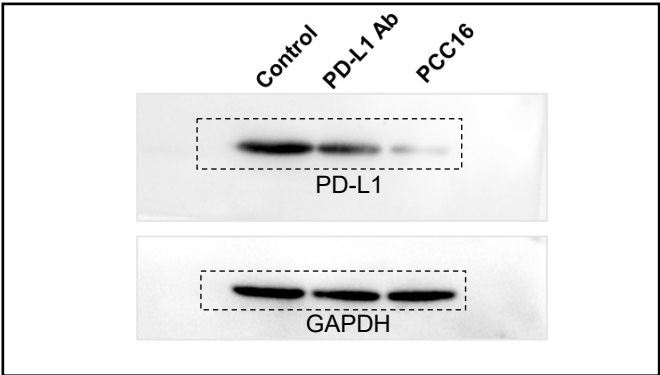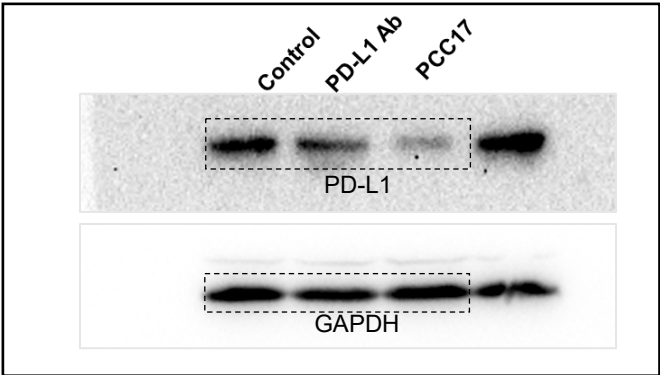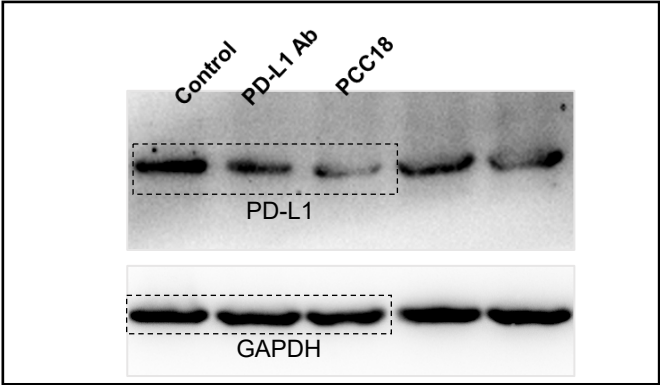

Figure 6a

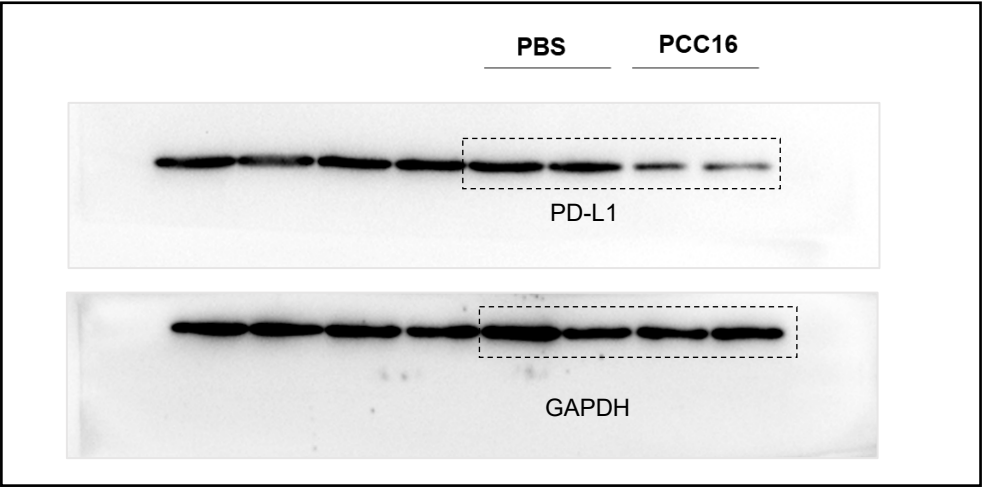

Figure 7h

Supplement: Supplementary file 2 — Source data of Werstern Blot [file 41419_2024_7073_MOESM2_ESM.pdf]
